# Apoptotic cell overload drives multinucleated giant cell formation after neoadjuvant chemotherapy in colorectal cancer: molecular mechanisms and tumor-suppressive effects

**DOI:** 10.1038/s41419-026-09320-w

**Published:** 2026-10-05

**Authors:** Jiaxin Xue, Zhiwei Wang, Xiaoliang Zhang, Sen Xu, Shuang Wu, Xiaoyue Sun, Meiqi Li, Xueyan Wu, Zhangming Chen, Ming Lu

**Affiliations:** 1 Department of Immunology, School of Basic Medical Sciences, Anhui Medical University, Hefei, China; 2 Department of Pathology, The First Affiliated Hospital of USTC, Division of Life Sciences and Medicine, University of Science and Technology of China, Hefei, China; 3 Department of Human Anatomy, School of Basic Medical Sciences, Anhui Medical University, Hefei, China; 4 Department of General Surgery, The First Affiliated Hospital of Anhui Medical University, Hefei, China

**Keywords:** Cell death and immune response, Tumour immunology

## Abstract

Colorectal cancer is a common digestive malignancy. Neoadjuvant chemotherapy (NAC) can downstage tumors and increase R0 resection rates. In colorectal cancer specimens with favorable pathological regression after NAC, we observed CD68^+^ multinucleated giant cells (MGCs) preferentially localized in apoptotic cell-rich areas, whereas they were rarely detected in non-NAC specimens. However, the mechanisms driving MGC formation and their functional significance remain unclear. We established an in vitro apoptotic cell-macrophage co-culture system to assess how apoptotic cell burden and efferocytosis regulate macrophage fusion. The TREM2/TYROBP signaling axis was examined using molecular and pharmacological approaches. RNA sequencing, pathway enrichment, lysosome- and metabolism-related transcriptional analyses, and secretory profiling were performed to characterize efferocytosis-induced MGCs. Their effects on colorectal cancer cells and the tumor microenvironment were further evaluated in vitro and in tumor-bearing mouse models. Excessive apoptotic cell burden markedly promoted macrophage fusion and upregulated the fusion-related molecules DCSTAMP/Dcstamp and CDH1/Cdh1. Apoptotic cell overload shifted macrophages from a homeostatic/efferocytic state toward a fusion-prone MGC phenotype. *TYROBP*/*Tyrobp* knockdown or loss inhibited macrophage fusion. Mechanistically, apoptotic cell overload activated the TREM2/TYROBP-MAPK/c-FOS-DCSTAMP axis, thereby enhancing *DCSTAMP* transcription and macrophage fusion. Efferocytosis-induced MGCs showed reduced lysosome-related transcriptional programs and an altered secretory profile, including increased TNF-α expression. Functionally, MGCs inhibited colorectal cancer cell growth in vitro. In tumor-bearing mice, MGCs reduced CD206⁺ macrophage and regulatory T-cell abundance while increasing CD8^+^ T-cell infiltration, thereby contributing to tumor growth suppression. Together, these findings identify efferocytosis-induced MGCs as a distinct macrophage state with altered lysosomal and secretory programs, anti-tumor activity, and the potential to remodel the tumor microenvironment.

## Introduction

In selected patients with locally advanced colorectal cancer, oxaliplatin-based fluoropyrimidine regimens (FOLFOX/CAPOX) can be used as neoadjuvant or perioperative treatment strategies to achieve tumor downstaging and improve the R0 resection rate [1, 2]. However, pathological responses to neoadjuvant chemotherapy (NAC) vary markedly among patients, and the host- and tumor-related determinants underlying this heterogeneity remain incompletely understood. The tumor microenvironment (TME), particularly tumor-associated macrophages (TAMs), has been implicated in shaping chemotherapy response. In the colorectal cancer specimens showing favorable pathological regression after NAC followed by curative resection, we observed CD68^+^ multinucleated giant cells (MGCs) preferentially localized near apoptotic cell-rich regions; by contrast, such cells were rarely detected in colorectal cancer samples resected without prior NAC. Successful NAC has been reported to induce substantial tumor-cell apoptosis in multiple cancer types [3, 4]. We also observed MGCs in a limited number of other tumors with favorable NAC responses, broadly consistent with previous descriptions of post-treatment pathological alterations in colorectal cancer [5–7] and other malignancies [8–14]. These observations prompted us to investigate the mechanisms underlying MGC formation in post-treatment apoptotic niches and their potential roles in apoptotic cell clearance, tissue remodeling, and therapeutic response.

Under physiological conditions, the body generates tens of millions of apoptotic cells each day [15]. Macrophages recognize phosphatidylserine (PS) exposed on apoptotic cells and engulf them through efferocytosis, thereby preventing secondary necrosis and limiting tissue damage while acquiring an anti-inflammatory, pro-repair immunoregulatory state that supports tissue homeostasis [16, 17]. In other settings, including infection, bone resorption, and foreign body responses, macrophages can undergo cell fusion and form MGCs [18]. These multinucleated cells differ in their mechanisms of formation, morphology, nuclear organization, and biological function. In atherosclerotic plaques, MGCs contribute to the handling of lipids, cholesterol crystals, and cellular debris, but may also exacerbate inflammation and promote plaque instability [19–21]. In the context of tumor nanotherapy, nanoparticle-induced MGCs have been reported to exert anti-tumor effects [22]. Together, these findings suggest that macrophage fusion into MGCs may reshape the TME and influence tumor progression. However, the impact of MGCs arising under conditions of excessive apoptotic-cell burden remains unclear.

TYROBP, also known as DAP12 or KARAP, is expressed in monocytes, macrophages, dendritic cells, and osteoclasts [23–25]. Previous studies have shown that the TREM2-TYROBP signaling axis upregulates the level of fusion molecule DC-STAMP (DCSTAMP), thereby mediating IL-4-induced MGC formation; this axis also participates in receptor activator of nuclear factor-κB ligand (RANKL)-driven osteoclast formation [18, 26]. In RANKL-RANK signaling, NF-κB and NFATc1 are coordinately activated, and nuclear NFATc1 induces the transcription of fusion-related proteins, including DC-STAMP and E-cadherin (CDH1) [18,26].

Beyond its role in fusion, TYROBP also participates in efferocytic and phagocytic receptor signaling [27, 28]. TREM2 promotes efferocytosis through coupling with TYROBP [29], and TYROBP is also capable of mediating macrophage fusion into MGCs [18, 26], we hypothesized that, under conditions of increased apoptotic burden and sustained efferocytic signaling, the TREM-TYROBP-associated pathway may coordinate both clearance-associated and fusion-associated programs, thereby promoting macrophage fusion into MGCs. However, the molecular mechanisms and functional state of MGCs generated in response to large-scale apoptotic-cell clearance remain poorly defined.

In this study, we established an in vitro apoptotic cell-macrophage co-culture system to quantitatively assess how apoptotic cell burden and efferocytosis regulate macrophage fusion. We then focused on the TYROBP-associated signaling axis and used molecular and pharmacological approaches to define its role in efferocytosis-driven MGC formation. To characterize the transcriptional and functional features of efferocytosis-induced MGCs, we performed RNA-sequencing (RNA-seq) with pathway enrichment analyses, analyzed metabolism- and lysosome-related transcriptional programs, profiled their secretory features, and explored how these changes might influence tumor-cell phenotypes and the TME. Finally, we explored the effects of MGCs on TME in a tumor-bearing model. Collectively, this study defines key pathway features associated with efferocytosis-linked MGC formation and suggests that the TYROBP-associated axis may contribute to post-treatment apoptotic-cell handling, TME remodeling, and tumor suppression.

## Materials and methods

### Cell culture and in vitro MGCs induction assay

THP-1 cells (3 × 10^5^ cells per well) (Procell, Wuhan, China), with half labeled with PKH26 (#D0030, Solarbio, Beijing, China) and the other half labeled with CFSE (#150347-59-4, AbMole, Shanghai, China), were cultured in Roswell Park Memorial Institute 1640 medium (RPMI 1640) (#350-000-CL, WISENT, Nanjing, China) supplemented with 1% 100× penicillin and streptomycin (#C002, Beyotime, Shanghai, China) and 10% fetal bovine serum (FBS, #086-150, WISENT, Nanjing, China). Cells were seeded into 24-well plates and stimulated with 50 ng/mL 12-O-tetradecanoyl phorbol-13-acetate (PMA, #N2060, APExBIO, Boston, USA) for 48 h to induce M0 differentiation.

RAW264.7 cells (Servicebio, Wuhan, China), peritoneal macrophages (PMs), and bone marrow-derived macrophages (BMDMs) (3 × 10^5^ cells per well), with half labeled with PKH26 and the other half labeled with CFSE, were cultured in Dulbecco’s Modified Eagle Medium (DMEM, #319-005-CL, WISENT, Nanjing, China) supplemented with 1% 100× penicillin and streptomycin and 10% FBS, and seeded into 24-well plates.

Jurkat cells (Procell, Wuhan, China) were exposed to UV irradiation (254 nm) for 15 min and then cultured in serum-free RPMI 1640 medium for 3 h at 37 °C in 5% CO_2_ to induce apoptosis. Apoptotic Jurkat cells were subsequently co-cultured with macrophages.

Mouse thymocytes were treated with 5 μM Dexamethasone (#BL171A, Biosharp, Beijing, China) in serum-free RPMI 1640 medium for 6 h at 37 °C in 5% CO_2_ to induce apoptosis. Apoptotic mouse thymocytes were subsequently co-cultured with macrophages.

For MerTK inhibition, THP-1-derived M0 macrophages were treated with UNC569 (40 nM) and then co-cultured with apoptotic cells at a 1:5 ratio for 4 h. Macrophage fusion was quantified by CFSE/PKH26 colocalization, and MAPK phosphorylation and DCSTAMP expression were assessed by western blotting.

### In vitro efferocytosis assay

Jurkat cells were exposed to UV irradiation as described above and co-cultured with Hoechst-stained THP-1 M0 cells for 2 h. Hoechst dye was purchased from YEASEN (#40731ES10, Shanghai, China).

### Mice

C57BL/6 wild-type (WT) and *Tyrobp*^*−/−*^ mice (male, 6–8 weeks old) were obtained from Cyagen Biosciences (Guangzhou, China) and housed in a specific pathogen-free facility under controlled conditions (20–25 °C, 12 h light/12 h dark cycle) with free access to food and water. After acclimatization at Anhui Medical University, mice were randomly assigned to experimental groups in a blinded manner where applicable. Investigators assessing outcomes were blinded to group assignments. Mice were monitored daily for post-operative or post-induction signs, including body weight, activity, and wound integrity. No unexpected adverse events occurred. All procedures were approved by the Experimental Animal Ethics Committee of Anhui Medical University (No. LLSC20231324).

### Human specimens and ethics approval

This study was conducted in accordance with the principles of the Declaration of Helsinki. The use of clinical pathological specimens in this study was reviewed and approved by the Biomedical Ethics Committee of Anhui Medical University (No. 83230484). Archival formalin-fixed paraffin-embedded (FFPE) tissue blocks were retrieved from the Department of Pathology of the Affiliated Hospital of the University of Science and Technology of China. The requirement for informed consent was waived by the ethics committee because this retrospective study used de-identified archival specimens and posed no more than minimal risk to patients. All specimens were anonymized before analysis to protect patient confidentiality.

### Quantitative real-time polymerase chain reaction (qRT-PCR)

Total RNA was extracted using total RNA extraction reagent (#YY101, Epizyme, Shanghai, China) and reverse-transcribed into cDNA using Hifair^®^ III 1st Strand cDNA Synthesis SuperMix (#11141ES60, YEASEN, Shanghai, China). qRT-PCR was performed using Hieff® qPCR SYBR Green Master Mix (#11201ES50, YEASEN, Shanghai, China) on a LightCycler 96 system (Roche, Switzerland). Relative gene expression was calculated using the 2^−ΔΔCt^ method and normalized to *GAPDH*. Primer sequences are listed in Supplementary Table S1.

### Western blot analysis

Cells were lysed in radio-immunoprecipitation assay (RIPA) lysis buffer (#IN-WB001, Invent, USA), and protein concentrations were determined using a BCA kit (#G2026, Servicebio, Wuhan, China). Equal amounts of protein were separated by SDS-PAGE and transferred onto PVDF membranes (Millipore, Billerica, MA, USA). After blocking, the membranes were incubated overnight at 4 °C with primary antibodies against AKT1/2/3 (#T55561, Abmart, Shanghai, China), phospho-AKT (#T40067, Abmart, Shanghai, China), PI3K (#T40115, Abmart, Shanghai, China), phospho-PI3K (#T40116, Abmart, Shanghai, China), TYROBP (#R383147, ZENBIO, Chengdu, China), ERK1/2 (#I04101864, Wanleibio, Shenyang, China), phospho-ERK1/2 (#I06281512, Wanleibio, Shenyang, China), p38 (#T55600, Abmart, Shanghai, China), phospho-p38 (#310091, ZENBIO, Chengdu, China), JNK (#R22866, ZENBIO, Chengdu, China), phospho-JNK (Thr183) (#381100, ZENBIO, Chengdu, China), DCSTAMP (#A14630, ABclonal, Wuhan, China), c-FOS (#66590-1-Ig, Proteintech, Wuhan, China), and β-actin (#20536-1-AP, Proteintech, Wuhan, China). Subsequently, membranes were incubated with the corresponding HRP-conjugated goat anti-rabbit IgG or HRP-conjugated goat anti-mouse IgG secondary antibodies (Proteintech, Wuhan, China). Chemiluminescent signals were detected using NcmECL High (#P2100, New Cell Molecular, Suzhou, China) and captured using a digital imaging system (Tanon, Shanghai, China).

### Stable knockdown of *TYROBP*

Lentiviral particles carrying shNC, sh*TYROBP*-1, sh*TYROBP*-2, and sh*TYROBP*-3 were generated by GENERAL BIOL (Chuzhou, China) and used to transduce THP-1 cells in the presence of HitransG A (#REVG004, GeneChem, Shanghai, China). After 72 h of culture at 37 °C, the medium was replaced with fresh medium. Starting 72 h post-infection, cells were selected with 2 µg/mL puromycin (#BL528A, Biosharp, Hefei, China) for 48 h and then maintained in fresh medium. The shRNA sequences are listed in Supplementary Table S2.

### Luciferase assay

A c-FOS plasmid (Miaolingbio, Wuhan, China) was transfected into HEK293T cells, followed by co-transfection with pGL3 reporter vectors (#E1751, Promega, Madison, USA) containing different promoter regions (pGL3-Basic, pGL3-1000, pGL3-500, and pGL3-Mut) and the pRL-TK vector (#E2241, Promega, USA) using PEI 40 K (#G1802, Servicebio, Wuhan, China). After 48 h, luciferase activities were measured using a luminometer (Promega, Madison, USA) together with the Luc-Pair™ Duo-Luciferase HS Assay Kit (#LF004, GeneCopoeia, USA). Firefly luciferase activity was normalized to Renilla luciferase activity.

### Immunofluorescence (IF)

Cells were fixed with 4% paraformaldehyde (#G1101, Servicebio, Wuhan, China) for 30 min, permeabilized with 0.5% Triton X-100 (#MS4311, Maokangbio, Shanghai, China) at room temperature for 20 min, and blocked with 10% goat serum for 1 h. Cells were then incubated overnight at 4 °C with primary antibodies diluted 1:100, followed by incubation with fluorophore-conjugated secondary antibodies (#B0801, Immunoway, Beijing, China) diluted 1:200 for 1 h at room temperature. Hoechst (#40731ES10, YEASEN, Shanghai, China) was used to stain nuclei before the addition of apoptotic cells. Confocal images were acquired using an FV3000 confocal microscopy system.

### siRNA knockdown

THP-1 cells were stimulated with 50 ng/mL PMA for 48 h to induce M0 polarization and then transfected with specific siRNAs using Advanced DNA RNA Transfection Reagent (#AD600075, Zeta Life, USA) according to the manufacturer’s instructions. The siRNAs were customized by Tsingke and General Biol. The siRNA sequences used in this study are listed in Supplementary Table S2.

### RNA-seq

For RNA-seq analysis, THP-1 M0 cells were co-cultured with apoptotic mouse thymocytes, and RAW264.7 cells were co-cultured with apoptotic Jurkat cells for 4 h. After co-culture, residual apoptotic cells were removed, and the remaining cells were lysed in the total RNA extraction reagent for RNA isolation. In experiments involving *DCSTAMP* knockdown, THP-1 M0 cells were transfected with siRNA against *DCSTAMP* before the addition of apoptotic mouse thymocytes, followed by co-culture and removal of residual apoptotic cells prior to RNA extraction. The siRNAs used in this study are listed in Supplementary Table S2. RNA-seq results are provided in Supplementary Tables S3–S6. To minimize transcript contamination from engulfed apoptotic cargo during RNA-seq, cross-species apoptotic targets were used in the human and mouse macrophage systems.

### Osteoclast induction

Bone marrow cells were harvested from the tibiae and femora of 6- to 8-week-old WT C57BL/6J mice and cultured overnight in α-MEM (#6125516, Gibco, USA) supplemented with 10% FBS and 1% P/S at 37 °C in 5% CO_2_. Non-adherent cells were collected and seeded into 24-well plates at 8 × 10^5^ cells per well. Osteoclastogenesis was induced with 40 ng/mL macrophage colony-stimulating factor (M-CSF) (#416-ML, R&D Systems, USA) and 40 ng/mL RANKL (#462-TEC, R&D Systems, USA). The medium was refreshed on day 5 with fresh cytokine-containing medium. Cells were fixed on day 6 or day 7 for subsequent analysis.

### Giemsa and TRAP staining

For Giemsa staining, cells were fixed according to the manufacturer’s instructions and incubated with diluted Giemsa stain (#C0131, Beyotime, Shanghai, China) for 35 min, followed by three washes with distilled water before imaging.

For tartrate-resistant acid phosphatase (TRAP) staining, cells were fixed according to the manufacturer’s instructions and incubated with TRAP staining solution (#G1492, Solarbio, Beijing, China) at 37 °C for 60 min according to the manufacturer’s instructions. Cells were then washed three times with distilled water before imaging.

### ELISA

The protein levels of TNF-α/Tnf-α in the culture supernatants of THP-1 cells and RAW264.7 cells were measured according to the manufacturers’ instructions using ELISA kits (#1117202, DAKEWE, Shenzhen, China; #YX-E20220M, Sinobestbio, Shanghai, China).

### CCK-8 assay for cell viability

Cell viability was assessed by CCK-8 assay. Briefly, cells (8 × 10^4^/well) were plated in 96-well plates and incubated with apoptotic cells for 4 h. CCK-8 reagent (10 µL) (#100-120, GOONIE, Guangzhou, China) was added and incubated for 1 h. Absorbance was recorded at 450 nm. The relative cell viability was normalized to the control group.

### Lactate dehydrogenase (LDH) release assay

Cytotoxicity was evaluated by measuring lactate dehydrogenase (LDH) release in the culture supernatants of cells using an LDH cytotoxicity assay kit (#A020-2, Nanjing Jiancheng Bioengineering Institute, Nanjing, China) according to the manufacturer’s instructions.

### Apoptosis assay

Following co-culture with apoptotic cells at the indicated macrophage:apoptotic-cell ratios for 4 h, macrophages were collected and stained using Annexin V-FITC/PI Apoptosis Detection Kit (#BB-4101, Bestbio, Nanjing, China) according to the manufacturer’s instructions. Samples were acquired on a BD Fortessa flow cytometer (BD Biosciences, USA) and analyzed using FlowJo v10. Apoptotic cells were defined as Annexin V^+^PI^+^ and Annexin V^+^PI^−^, and three independent experiments were analyzed.

### Chromatin immunoprecipitation (ChIP) assay

ChIP assays targeting the promoter region were performed using an Enzymatic Chromatin IP Kit (#9005, CST, MA, USA). Lysates were sonicated and further digested with micrococcal nuclease to generate chromatin fragments. Anti-c-FOS antibody (#2250, CST, MA, USA) was used for immunoprecipitation, and normal rabbit IgG (#2729, CST, MA, USA) was used as the negative control. Primer sequences used in this study are listed in Supplementary Table S1.

### Single-cell RNA-seq analysis of therapy-associated fusion-like myeloid/macrophage populations

Publicly available single-cell RNA-seq count matrices and corresponding metadata for GSE178318 [30], GSE254249 [31], and GSE280311 [32] were downloaded from the NCBI Gene Expression Omnibus (GEO). For GSE178318, the analysis was restricted to myeloid-lineage cells from primary colorectal tumors and colorectal liver metastases obtained from patients with or without preoperative chemotherapy; peripheral blood samples were excluded. For GSE254249, the analysis was restricted to myeloid-lineage cells from rectal tumor tissues collected before and after neoadjuvant therapy; peripheral blood samples were excluded. GSE280311 was used as a colorectal tumor macrophage-state reference for subtype comparison. Publicly available colorectal cancer single-cell RNA-seq datasets EMTAB8107 and GSE166555 were examined using the Tumor Immune Single-cell Hub 2 (TISCH2) database.

Single-cell RNA-seq data from GSE178318 and GSE254249 were analyzed using Seurat [30, 31, 33]. Myeloid-lineage cells were retained based on the original annotations and canonical myeloid marker expression, followed by log-normalization, identification of highly variable genes, scaling, principal component analysis, graph-based clustering, and UMAP visualization [33]. Treatment-enriched myeloid clusters were identified according to the proportion and abundance of post-treatment cells within each cluster, and cluster identities were assigned using differentially expressed marker genes relative to other myeloid cells.

To evaluate fusion-like macrophage features, we calculated a fusion score using a curated gene set including *DCSTAMP*, *OCSTAMP*, *ATP6V0D2*, *CDH1*, *CD9*, *CD44*, *SIRPA*, *ADAM8*, *ADAM9*, *ADAM12*, *MMP9*, *MMP14*, *ITGAM*, *ITGB2*, *ITGAX*, *TYROBP*, and *TREM2* [18, 34, 35]. In this gene set, *DCSTAMP*, *OCSTAMP*, and *ATP6V0D2* represent canonical macrophage/osteoclast-like fusion-associated genes; *CDH1*, *CD9*, *CD44*, *SIRPA*, *ITGAM*, *ITGB2*, and *ITGAX* reflect cell-cell adhesion, macrophage interaction, phagocytic synapse formation, and membrane contact programs; *ADAM8*, *ADAM9*, *ADAM12*, *MMP9*, and *MMP14* represent membrane protein shedding, extracellular matrix remodeling, and tissue remodeling; and *TYROBP* and *TREM2* reflect macrophage activation, phagocytosis, lipid/damage sensing, and immunoregulatory signaling. Only genes detected in each dataset were used for scoring, and per-cell module scores were calculated using the Seurat AddModuleScore function [36].

MAPK activity was evaluated using a canonical MAPK activation score based on MAPK/ERK immediate-response and negative-feedback genes, including *DUSP6, DUSP4, DUSP5, SPRY2, SPRY4, SPRED1, SPRED2, ETV1, ETV4, ETV5, FOS, FOSB, FOSL1, JUN, JUNB, JUND, EGR1, EGR2, EGR3, IER2, IER3, CCND1, AREG, EPHA2, PHLDA1, PHLDA2*, and *ELK3* [37–39]. Among these genes, *FOS, FOSB, FOSL1, JUN, JUNB, JUND, EGR1, EGR2, EGR3, IER2*, and *IER3* represent immediate-early transcriptional responses rapidly induced downstream of MAPK/ERK signaling; *DUSP4, DUSP5, DUSP6, SPRY2, SPRY4, SPRED1*, and *SPRED2* represent negative-feedback regulators induced by MAPK activation to restrain ERK signaling; *ETV1, ETV4, ETV5*, and *ELK3* reflect ETS-family transcriptional output downstream of ERK; and *CCND1, AREG, EPHA2, PHLDA1*, and *PHLDA2* represent MAPK-associated downstream programs related to proliferation, epithelial-stromal signaling, receptor signaling, and stress adaptation. Differences in fusion score and MAPK activation score between treatment-naive and treated cells were tested using the Wilcoxon rank-sum test, with Benjamini–Hochberg correction for multiple comparisons [40].

To compare candidate clusters with the in vitro MGC transcriptional program, cluster-upregulated genes were defined by differential expression analysis using log_2_FC > 0.585 and FDR < 0.05, followed by hypergeometric enrichment analysis against the in vitro MGC-upregulated gene set. Candidate populations were further compared with GSE280311 colorectal cancer macrophage reference subtypes [32] by calculating subtype marker module scores. Visualization was performed using ggplot2 [41].

### Tumor-bearing mouse model

MC38 cells (1 × 10^6^ cells per mouse) (Servicebio, Wuhan, China) suspended in PBS were injected subcutaneously into the right dorsal flank of 6-week-old male C57BL/6 WT mice. Seven days after injection, when primary tumors reached approximately 50–100 mm^3^, MGCs and M0 derived from RAW264.7 cells were administered via intratumoral injection every 3 days over a 2-week period. Each mouse received 1 × 10^6^ cells suspended in 50 μL of PBS.

To assess the requirement for TYROBP in therapy-associated MGC formation in vivo, age- and sex-matched wild-type and *Tyrobp*^*−/−*^ C57BL/6 mice bearing subcutaneous MC38 tumors were treated with oxaliplatin (5 mg/kg, intraperitoneally) (#M2290, AbMole, USA) according to the following schedule: once every 2 days, for a total of seven times. Tumors were collected at day 21, and CD68^+^ multinucleated cells and tumor-cell apoptosis were assessed by immunofluorescence. Each group contained six mice.

### Co-culture assay

MC38 and HT-29 cells (2 × 10^4^ per well) were seeded into six-well plates and cultured with conditioned medium (CM) collected from MGCs or M0 cells for 4–5 days.

### Flow cytometry

Tumor tissues were collected after anesthesia with isoflurane. The tissues were mechanically dissociated and incubated in DMEM containing 1 mg/mL collagenase IV (#2091MG100, BioFroxx, Germany) at 37 °C for 30 min. After filtration through a 70 μm cell strainer, tumor cells were removed by density gradient centrifugation (750 × *g* for 30 min) using 30% and 70% Percoll (#P8370, Solarbio, Beijing, China). Single-cell suspensions were resuspended in 1% BSA and stained with the following antibodies: anti-mouse-CD8a-FITC (#100705, Biolegend, San Diego, USA), anti-mouse-CD3-PE (#100205, Biolegend, San Diego, USA), anti-mouse-CD4-APC (#100412, Biolegend, San Diego, USA), anti-mouse-FOXP3-Pacific Blue (#126410, Biolegend, San Diego, USA), anti-mouse-CD45-PerCP/Cyanine5.5 (#103132, Biolegend, San Diego, USA), anti-mouse/human-CD11b-APC/Cyanine7 (#101226, Biolegend, San Diego, USA), anti-mouse-Ly6C-FITC (#128005, Biolegend, San Diego, USA), anti-mouse-CD206-PE (#141705, Biolegend, San Diego, USA), Zombie UV^TM^ Dye (#423107, Biolegend, San Diego, USA), and anti-mouse-F4/80-Brilliant Violet 510^TM^ (#123135, Biolegend, San Diego, USA). Data were acquired on a BD Fortessa flow cytometer (BD Biosciences, USA) and analyzed using FlowJo v10 software.

### Statistical analyses

Data were analyzed using GraphPad Prism 10.0. Results are presented as mean ± SD. Comparisons between two groups were performed using unpaired Student’s *t* tests, while comparisons among multiple groups were performed using one-way ANOVA followed by Dunnett’s post hoc test or two-way ANOVA followed by Dunnett’s post hoc test, as appropriate. Statistical significance was defined as follows: *p < 0.05, **p < 0.01, and ***p < 0.001.

## Results

### NAC induces multinucleated giant macrophages in apoptosis-enriched regions, and efferocytosis drives their formation in vitro

**Fig. 1:**
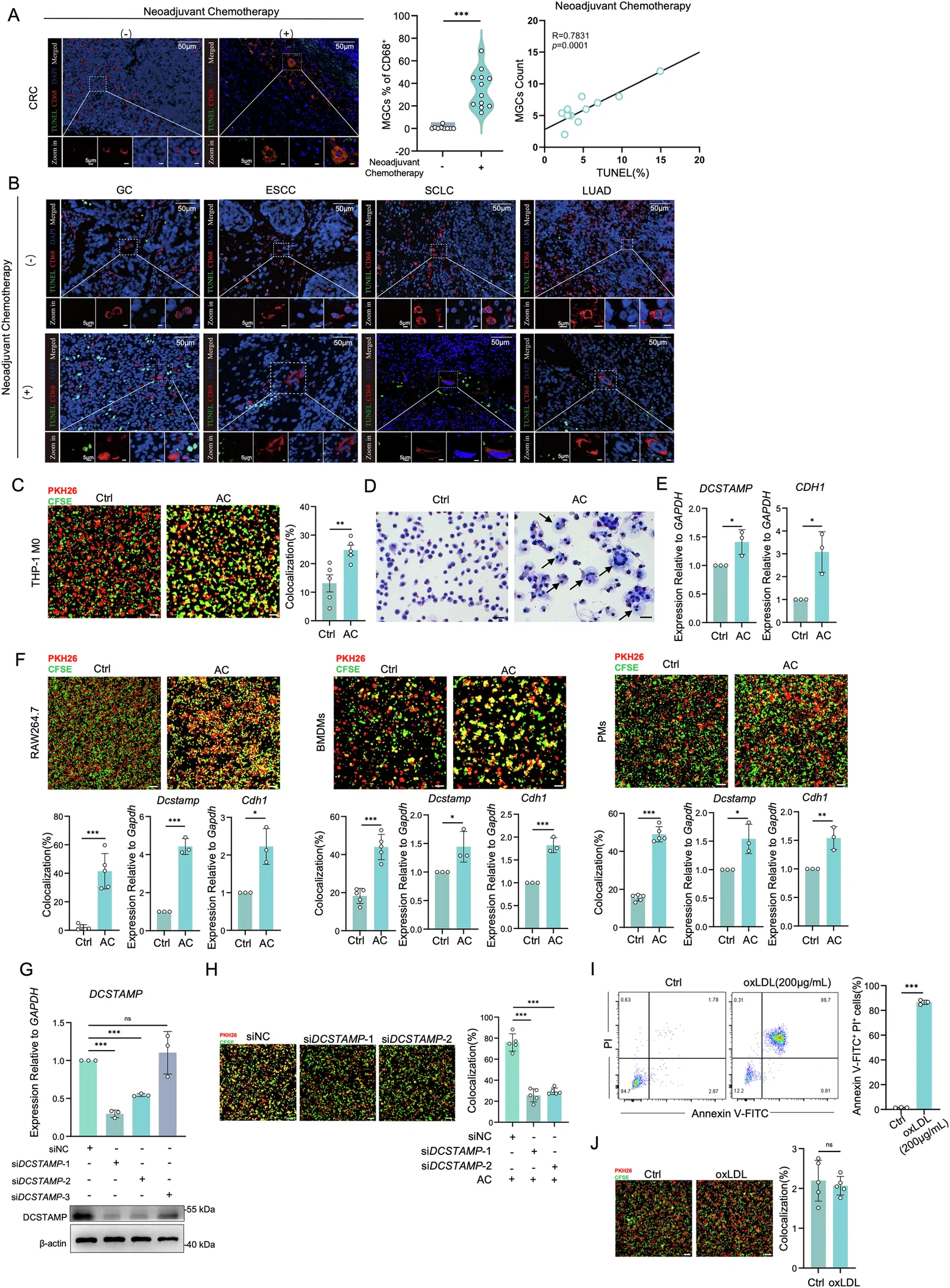
Apoptotic cells promote the formation of MGCs by macrophages. **A** Immunofluorescence analysis and correlation analysis of CD68^+^ MGCs and apoptosis in human CRC tissues (*n*(NAC^−^) = 9, *n*(NAC^+^) = 12). Scale bars: 50 μm and 5 μm. **B** Immunofluorescence analysis of MGCs in tumor tissue from human. Scale bars: 50 μm and 5 μm. **C** THP-1-derived M0 were labeled with CFSE and PKH26, as control group (Ctrl), and fusion was induced by exposure to apoptotic cells (AC). Macrophage fusion is represented by colocalization of the red and green fluorescent labels (yellow) (*n* = 5 per group). Scale bar, 100 μm. **D** Giemsa staining of THP-1-derived M0 and MGCs. Scale bar, 50 μm. **E** Quantitative PCR analysis of *DCSTAMP* and *CDH1* in Ctrl and AC groups (*n* = 3 per group). **F** RAW264.7 cells, BMDMs and PMs were labeled with CFSE and PKH26, as control group, and fusion was induced by exposure to AC (*n* = 5 per group). Scale bar, 100 μm. Quantitative PCR analysis of *DCSTAMP* and *CDH1* (lower right) (*n* = 3 per group). **G** Quantitative PCR and Western blot analysis of the mRNA and protein knockdown levels of DCSTAMP in THP-1-derived M0 (*n* = 3 per group). **H** THP-1-derived M0 with or without *DCSTAMP* knockdown were labeled with CFSE and PKH26 and fusion was induced by exposure to AC (*n* = 5 per group). Scale bar, 100 μm. **I** FACS analysis of necrosis in Jurkat cells following treatment with 200 μg/mL oxidized low-density lipoprotein (oxLDL) for 48 h (*n* = 3 per group). Annexin V/PI double-positive cells represent secondary necrosis. **J** Macrophage fusion of THP-1-derived M0 cells following co-culture with necrotic Jurkat cells treated with 200 μg/mL oxidized low-density lipoprotein (oxLDL) for 48 h (*n* = 5 per group). Scale bar, 100 μm. Mean ± SD, *p < 0.05, **p < 0.01, ***p < 0.001.

In patients with locally advanced CRC, NAC is commonly used to downstage or shrink tumors and thereby improve the rate of curative resection. We performed anti-CD68 immunostaining and TUNEL staining on tumor tissues from 12 CRC patients who underwent surgical resection after NAC and 9 CRC patients who underwent upfront surgery without prior chemoradiotherapy. We found that multiple CD68^+^ MGCs were present in CRC tissues after NAC, and these cells were preferentially observed in close proximity to TUNEL^+^ cells, and their abundance was positively correlated with the number of surrounding TUNEL^+^ cells. By contrast, such MGCs were rarely observed in untreated CRC tissues (Fig. 1A). MGCs were defined as CD68^+^ cells with continuous cytoplasm containing ≥2 nuclei. These findings suggest that the increased apoptosis observed after NAC is associated with the formation of MGCs within tumor tissues. We further examined gastric cancer (GC), esophageal squamous cell carcinoma (ESCC), small cell lung cancer (SCLC), and lung adenocarcinoma (LUAD) tissues and found that MGCs were also more frequently observed in NAC-treated samples, in close proximity to TUNEL^+^ cells, whereas such changes were uncommon in samples without prior chemoradiotherapy (Fig. 1B). These observations suggest that MGC formation accompanied by increased apoptosis after NAC may represent a shared phenomenon across tumor types.

We next established an in vitro efferocytosis-fusion model in which THP-1-derived macrophages engulfed apoptotic Jurkat cells. Two macrophage populations were separately labeled with CFSE and PKH26 and then co-cultured; fused cells were identified as CFSE^+^PKH26^+^ double-positive cells (Fig. 1C). Multinucleated morphology was further confirmed by Giemsa staining (Fig. 1D). THP-1 macrophages underwent marked fusion and formed MGCs after efferocytosis (Fig. 1C, D), accompanied by increased expression of the fusion-related genes *DCSTAMP* and *CDH1* (Fig. 1E). Similar results were observed in RAW264.7 cells, mouse bone marrow-derived macrophages (BMDMs), and mouse peritoneal macrophages (PMs) (Fig. 1F). Knockdown of *DCSTAMP* significantly reduced apoptotic cell-induced macrophage fusion (Fig. 1G, H), indicating that DCSTAMP is also required for this fusion process [42]. Considering that, in addition to apoptosis, necrosis may also be present in tumors after NAC, in addition to apoptosis, we co-cultured macrophages with necrotic cells generated by oxLDL treatment (Fig. 1I). Necrotic cells failed to induce fusion in THP-1-derived M0 macrophages (Fig. 1J), suggesting that MGC formation in this setting is more consistent with a response induced by efferocytosis of apoptotic cells.

### Apoptotic cell burden determines whether macrophages undergo homeostatic efferocytosis or differentiate into fusion-prone MGCs

**Fig. 2:**
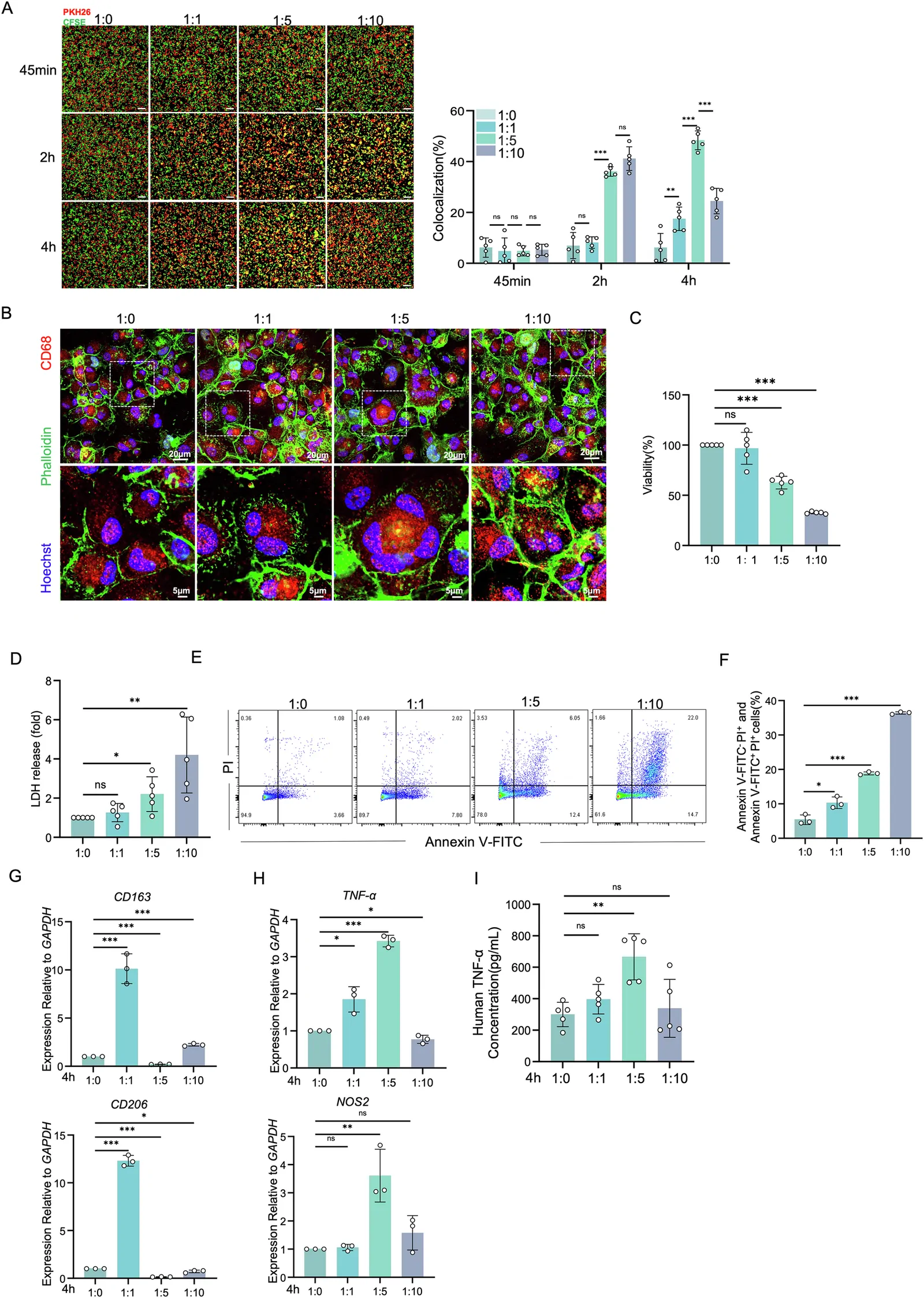
The timing and proportion of apoptotic cells and macrophages co-culture determine the formation of MGCs. **A** THP-1-derived M0 were labeled with CFSE and PKH26 and fusion was induced by exposure to AC at different ratios for different times. Macrophage fusion is represented by colocalization of the red and green fluorescent labels (yellow) (*n* = 5 per group). Scale bar, 100 μm. **B** Immunofluorescence analysis of MGCs formation in THP-1-derived M0 macrophages co-cultured with different ratios of AC. Data are shown as representative pictures. **C** CCK-8 analysis of cell viability in THP-1-derived M0 macrophages co-cultured with different ratios of AC (*n* = 5 per group). **D** LDH release analysis in THP-1-derived M0 macrophages co-cultured with various ratios of AC (*n* = 5 per group). **E** FACS analysis of apoptosis in THP-1-derived M0 macrophages co-cultured with different ratios of AC. **F** Statistical graph of apoptotic rate (*n* = 3 per group). Quantitative PCR analysis of CD163 and CD206 (**G**) and *TNF-α*, *NOS2* (**H**) (*n* = 3 per group). **I** ELISA analysis of TNF-α in THP-1-derived M0 and THP-1-derived M0 exposed to AC at different ratios (*n* = 5 per group). Mean ± SD, *p < 0.05, **p < 0.01, ***p < 0.001.

We systematically evaluated how the ratio of macrophages to apoptotic cells (Mφ:Apop) and the duration of co-culture influenced macrophage fusion. We found that fusion was most pronounced when THP-1-derived M0 macrophages were co-cultured with apoptotic Jurkat cells at a 1:5 ratio for 4 h in vitro (Fig. 2A, B), suggesting that both apoptotic-cell burden and stimulation time define an optimal window for MGC formation. This was consistent with an inverted U-shaped effect: MGC formation decreased when apoptotic-cell burden was either too low or too high, or when the co-culture period was either too short or too long. We subsequently found that, at an Mφ ratio of 1:10, macrophage viability was significantly reduced (Fig. 2C), accompanied by increased LDH release (Fig. 2D) and elevated apoptosis (Fig. 2E, F). These findings suggest that an excessive apoptotic-cell burden compromises macrophage survival, potentially accounting for the reduced MGC formation observed under these conditions.

Previous studies have shown that macrophage clearance of apoptotic cells, namely efferocytosis, usually promotes a shift toward an immunoregulatory or tissue-reparative state [43, 44]. Consistent with this notion, we found that when the Mφ:Apop ratio was 1:1, macrophages expressed higher levels of marker genes associated with immunoregulatory and reparative macrophage programs (Fig. 2G), suggesting a greater tendency toward a pro-resolving efferocytic state. In contrast, when the ratio was increased to 1:5, the expression of these immunoregulatory and reparative markers was significantly reduced (Fig. 2G), whereas inflammatory effector genes, such as *TNF-α* and *NOS2* were upregulated (Fig. 2H, I). Under the same 1:5 co-culture condition, increased expression of *Tnf-α* and *Nos2* was also observed in mouse RAW264.7 cells (Supplementary Fig. S1A).

These results suggest that apoptotic-cell burden and the duration of ongoing efferocytosis may be important determinants of whether macrophages preferentially adopt an immunoregulatory and pro-resolving state after efferocytosis or instead undergo fusion into MGCs accompanied by an inflammatory transcriptional profile.

### Apoptotic cell overload induces transcriptional upregulation of the macrophage MAPK stress-response program

**Fig. 3:**
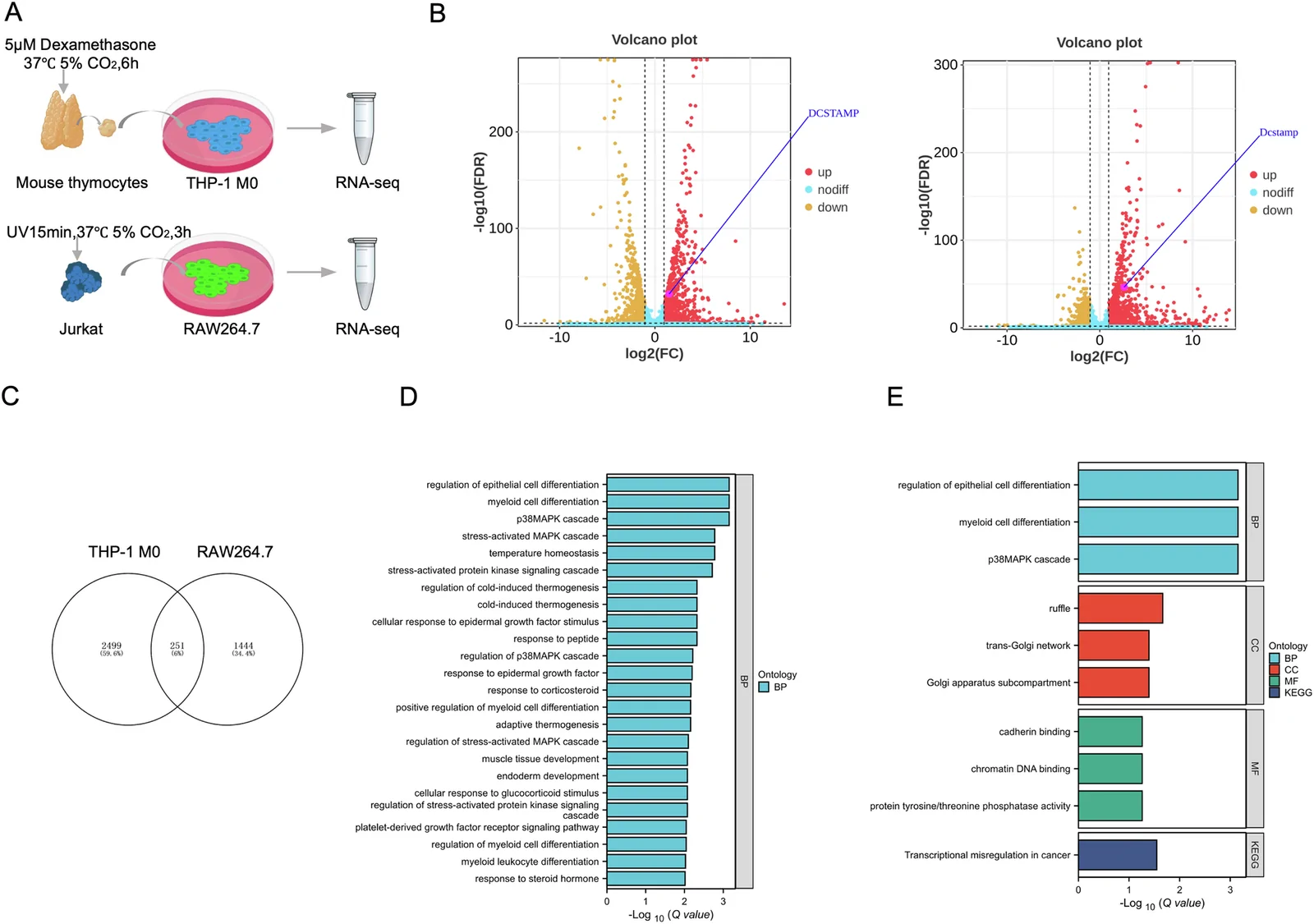
Transcriptomic profiling reveals activation of stress-responsive MAPK signaling and repair programs in apoptotic cell-induced MGCs. **A** Schematic overview of transcriptomic profiling and downstream analyses. **B** Volcano plot illustrating *DCSTAMP*/*Dcstamp* upregulation in macrophages following apoptotic cell-induced fusion. **C** Number of upregulated genes and co-upregulated genes in THP-1 M0 and RAW264.7 cells (*n* = 3 per group). Log_2_FC > 0.585, FDR < 0.05. **D**, **E** GO and KEGG enrichment analysis of commonly upregulated genes in human and mouse apoptotic cell-induced MGCs.

To comprehensively characterize the cellular state of efferocytosis-induced MGCs, we performed RNA-seq on fused macrophage populations generated after 4 h of co-culture with apoptotic cells under apoptotic-cell overload conditions (macrophage:apoptotic cell = 1:5). These included RAW264.7 cells co-cultured with apoptotic Jurkat cells and THP-1-derived M0 macrophages co-cultured with apoptotic mouse thymocytes, together with separately cultured control macrophages (Fig. 3A and Supplementary Tables S3 and S4). The results showed that *DCSTAMP*/*Dcstamp* was significantly upregulated in both human THP-1 macrophages and mouse RAW264.7 cells (Fig. 3B). We then performed GO/KEGG enrichment analysis on genes commonly upregulated (Log_2_FC > 0.585, FDR < 0.05) in both human and mouse macrophages systems (Fig. 3C and Supplementary Table S5). Among the top 24 enriched terms, upregulated genes were prominently enriched in “p38MAPK cascade” and “stress-activated MAPK cascade”, suggesting that under conditions of sustained extracellular stimulation and oxidative stress, both human and mouse macrophages activate a MAPK stress-response axis that may promote cytoskeletal remodeling, inflammatory regulation, and antioxidant responses [45–47] (Fig. 3D). Further analysis at the cellular component and molecular function levels revealed enrichment in terms such as “ruffle”, “trans-Golgi network”, “Golgi apparatus subcompartment”, “cadherin binding”, “chromatin DNA binding”, and “tyrosine/serine-threonine phosphatase activity”, consistent with enhanced phagocytic and secretory activity, increased adhesive/fusogenic capacity, and broad remodeling of transcriptional and signaling networks (Fig. 3E).

### The TYROBP-MAPK axis contributes to apoptotic cell-induced macrophage fusion independently of IL-4- and RANKL-driven programs

**Fig. 4:**
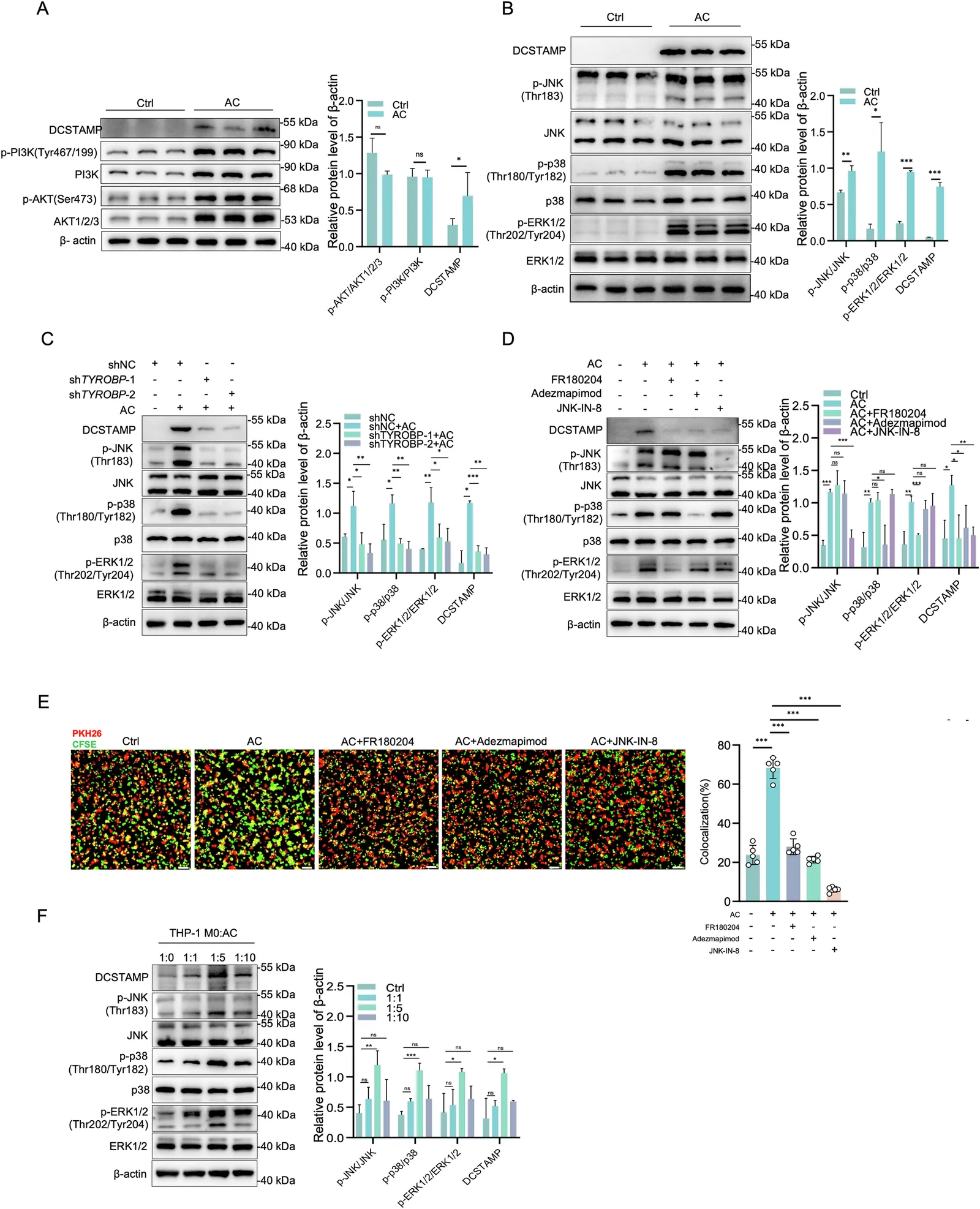
MAPK signaling mediates apoptotic cell-induced DCSTAMP upregulation and macrophage fusion. **A** Western blot analysis of the protein levels of DCSTAMP, p-PI3K, PI3K, p-AKT, AKT1/2/3 and β-actin (loading control) following co-culture with apoptotic cells (AC) (*n* = 3 per group). **B**–**D** Western blot analysis of the protein levels of DCSTAMP, p-JNK, JNK, p-p38, p38, p-ERK1/2, ERK1/2 and β-actin (loading control) in the indicated groups (*n* = 3 per group). **E** THP-1-derived M0 were labeled with CFSE and PKH26 and fusion was induced under control, AC, or AC plus 10 μM FR180204 or 1 μM adezmapimod or 50 nM JNK-IN-8 treatment. Macrophage fusion is represented by colocalization of the red and green fluorescent labels (yellow). Data are shown as representative pictures (left), and quantitation of colocalization (right) (*n* = 5 per group). Scale bar, 100 μm. **F** Western blot analysis of the protein levels of DCSTAMP, p-JNK, JNK, p-p38, p38, p-ERK1/2, ERK1/2 and β-actin (loading control) in THP-1-derived M0 exposed to AC at different ratios (*n* = 3 per group). Mean ± SD, *p < 0.05, **p < 0.01, ***p < 0.001.

Because RANKL-NFATc1 [48] and MAPK [49] are classical pathways regulating *DCSTAMP* transcription, and TYROBP can activate both the PI3K/AKT and MAPK pathways through Syk signaling [50], we examined the activation status of PI3K/AKT and MAPK signaling in macrophages after co-culture with apoptotic cells. We found that the PI3K/AKT axis was not significantly activated (Fig. 4A), whereas the MAPK pathway was robustly activated, accompanied by increased DCSTAMP expression (Fig. 4B). Notably, *TYROBP* knockdown markedly reduced both MAPK activation and DCSTAMP expression (Fig. 4C), suggesting that TYROBP serves as an important upstream node linking efferocytic input to the MAPK-DCSTAMP axis under conditions of apoptotic-cell overload. To assess the requirement for individual MAPK branches in macrophage fusion, we treated cells with the ERK inhibitor FR180204, the p38 inhibitor adezmapimod, and the JNK inhibitor JNK-IN-8. All three inhibitors suppressed *DCSTAMP* expression (Fig. 4D) as well as macrophage fusion (Fig. 4E), indicating that MAPK signaling contributes to macrophage fusion induced by apoptotic-cell overload. Further comparison of MAPK activation under different apoptotic-cell burdens showed that, consistent with the degree of fusion, MAPK activation was strongest at a macrophage:apoptotic cell ratio of 1:5 (Fig. 4F).

**Fig. 5:**
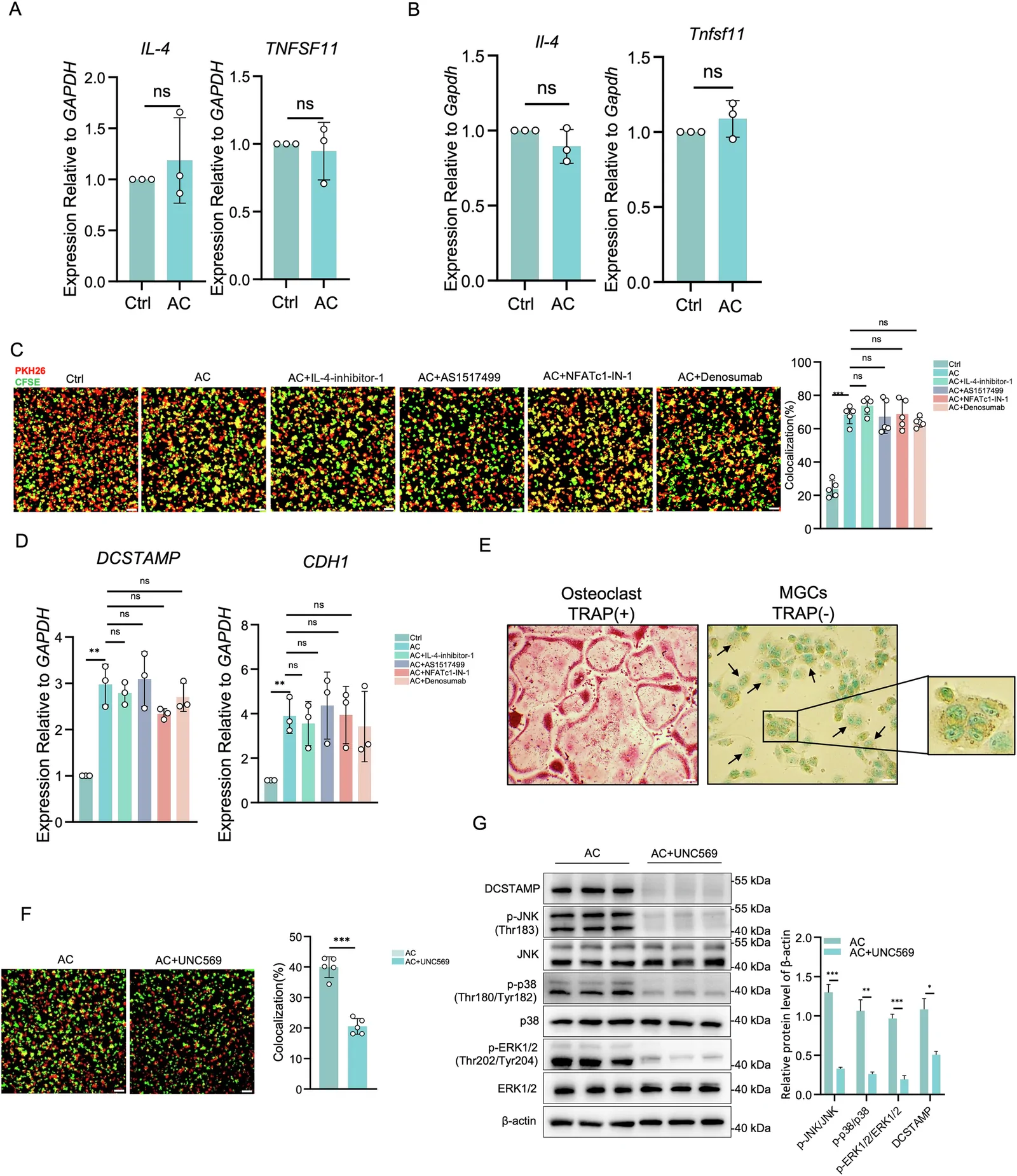
Apoptotic cell-induced macrophage fusion occurs independently of the IL-4-STAT6 and RANKL-NFATc1 pathways. **A**, **B** Quantitative PCR analysis of *IL-4*/*Il-4* and *TNFSF11*/*Tnfsf11* in Ctrl and AC groups (*n* = 3 per group). **C** THP-1-derived M0 were labeled with CFSE and PKH26 and fusion was induced under control, AC, or AC plus 5 μM IL-4-inhibitor-1 or 50 nM AS1517499 or 5 μM NFATc1-IN-1 or 2.5 μM denosumab treatment. Macrophage fusion is represented by colocalization of the red and green fluorescent labels (yellow). Data are shown as representative pictures, and quantitation of colocalization (*n* = 5 per group). Scale bar, 100 μm. **D** Quantitative PCR analysis of *DCSTAMP* and *CDH1* (*n* = 3 per group). **E** TRAP staining of THP-1-derived M0 exposed to AC. Data are shown as representative pictures. Scale bar, 50 μm. **F** Representative fluorescence images and quantification of AC-induced macrophage fusion with or without the MerTK inhibitor UNC569 (40 nM) (*n* = 5 per group). Scale bar, 100 μm. **G** Western blot and densitometric analyses of DCSTAMP and total and phosphorylated JNK, p38, and ERK1/2 following UNC569 treatment (*n* = 3 per group). β-actin served as the loading control. Small-molecule compound used in this study are listed in Supplementary Table S7. Mean ± SD, *p < 0.05, **p < 0.01, ***p < 0.001.

Because both IL-4 and RANKL can promote macrophage fusion [18], we next examined the expression of *IL4* and *RANKL*. Their expression levels did not change significantly in the presence or absence of apoptotic-cell co-culture (Fig. 5A, B). In the apoptotic cell co-culture system, treatment with the IL-4 inhibitor IL-4-inhibitor-1, the STAT6 inhibitor AS1517499, the RANKL-neutralizing antibody denosumab, or the NFATc1 inhibitor NFATc1-IN-1 did not significantly affect apoptotic cell-induced macrophage fusion (Fig. 5C) or *DCSTAMP*/*CDH1* expression (Fig. 5D). In addition, TRAP staining of apoptosis-induced MGCs was negative (Fig. 5E), suggesting that these cells did not undergo osteoclast-like differentiation. Taken together, these results indicate that apoptotic cell-induced macrophage fusion depends on the TYROBP-MAPK axis rather than on the classical IL-4-STAT6 or RANKL-NFATc1 programs.

Because apoptotic cells are recognized by multiple efferocytic receptors, some of which activate MAPK signaling, we asked whether receptors other than TREM2 contribute to macrophage fusion. We focused on MerTK, a canonical efferocytic receptor previously linked to MAPK activation [51]. Pharmacological inhibition of MerTK with UNC569 reduced apoptotic cell-induced macrophage fusion (Fig. 5F), attenuated JNK, p38, and ERK1/2 phosphorylation, and decreased DCSTAMP expression (Fig. 5G). These findings identify MerTK as an additional contributor to macrophage fusion and suggest that MerTK and TREM2–TYROBP may provide parallel inputs to MAPK activation.

### c-FOS directly binds to and transcriptionally activates DCSTAMP

**Fig. 6:**
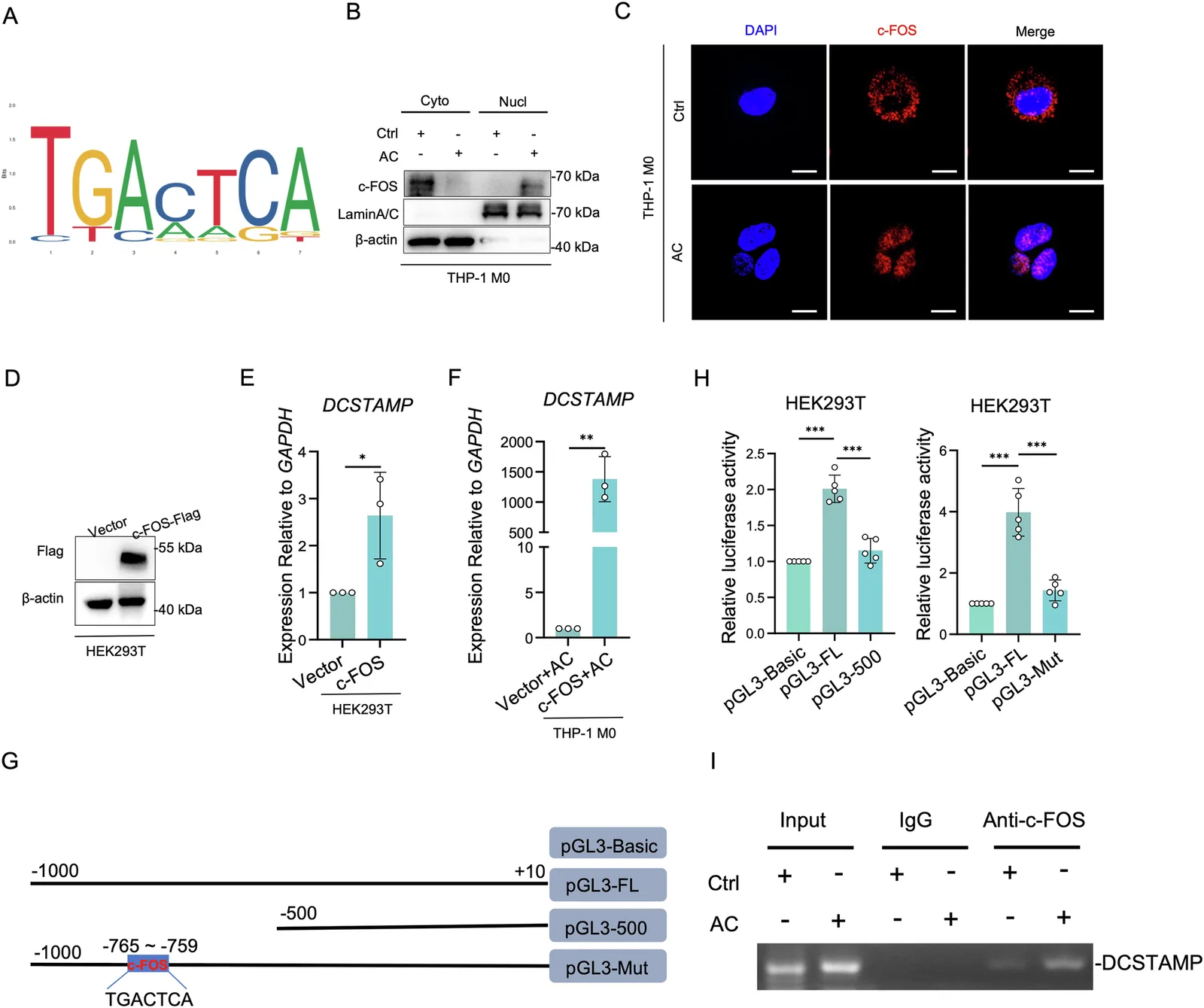
c-FOS functions as a transcription factor that upregulates DCSTAMP expression. **A** The JASPAR database predicted c-FOS binding sites on the DCSTAMP promoter. **B** Western blot analysis of the protein levels of c-FOS, LaminA/C (loading control) and β-actin (loading control) in cytoplasmic or nuclear fractions of THP-1-derived M0 with or without AC treatment. **C** Immunofluorescence analysis of c-FOS in THP-1-derived M0 with or without AC treatment. Data are shown as representative pictures. Scale bar, 20 μm. **D** Validation of c-FOS overexpression in HEK293T cells by western blotting. **E** Quantitative PCR analysis of *DCSTAMP* in HEK293T cells with or without c-FOS overexpression (*n* = 3 per group). **F** Quantitative PCR analysis of *DCSTAMP* in THP-1-derived M0 exposed to AC with or without c-FOS overexpression (*n* = 3 per group). **G** Schematic illustration of pGL3-basic plasmid construction with DCSTAMP promoter truncations. **H** Dual-luciferase reporter analysis of promoter activity was performed in HEK293T cells at 48 h post-transfection (*n* = 5 per group). **I** ChIP-PCR analysis of the c-FOS binding site. Mean ± SD, *p < 0.05, **p < 0.01, ***p < 0.001.

To identify transcription factors downstream of MAPK that promote DCSTAMP expression, we performed enrichment analysis based on ChEA/ChEA3, which suggested that c-FOS may bind to the DCSTAMP promoter region (Fig. 6A). Because MAPK signaling is known to promote c-FOS activation and nuclear translocation through phosphorylation [52, 53], we examined c-FOS localization in macrophages after co-culture with apoptotic cells and found a marked increase in nuclear c-FOS (Fig. 6B, C). Moreover, c-FOS overexpression in HEK293T cells enhanced *DCSTAMP* transcription (Fig. 6D, E), and *DCSTAMP* expression was further increased under efferocytic co-culture conditions (Fig. 6F).

Dual-luciferase reporter assays showed that the −1000 to −500 bp region of the DCSTAMP promoter, relative to the transcription start site (TSS), exhibited strong transcriptional activating activity, whereas deletion or mutation of the predicted AP-1/c-FOS binding motif within this region significantly reduced promoter activity (Fig. 6G, H). ChIP-PCR analysis further confirmed that c-FOS directly binds to the predicted binding site within the *DCSTAMP* promoter (Fig. 6I). Collectively, these findings support a model in which efferocytosis activates c-FOS downstream of TYROBP-mediated MAPK signaling, promoting its nuclear translocation and direct transcriptional activation of *DCSTAMP*, thereby facilitating macrophage fusion.

### Apoptotic cell overload-induced macrophage fusion involves efferocytosis-associated signaling and impaired apoptotic-cell degradation

When efferocytosis is continuously initiated and successfully completed, macrophages do not undergo fusion or acquire a pro-inflammatory phenotype, but instead develop an anti-inflammatory and reparative state. To determine which stage of the efferocytosis process participates in macrophage fusion induced by apoptotic-cell overload, we separately perturbed three stages of the efferocytic program: (1) recognition of phosphatidylserine (PS) by efferocytic receptors and initiation of signaling; (2) cytoskeletal rearrangement and phagocytic internalization; and (3) post-engulfment lysosomal degradation followed by transcriptional reprogramming toward immunoregulatory and tissue-reparative programs [17].

**Fig. 7:**
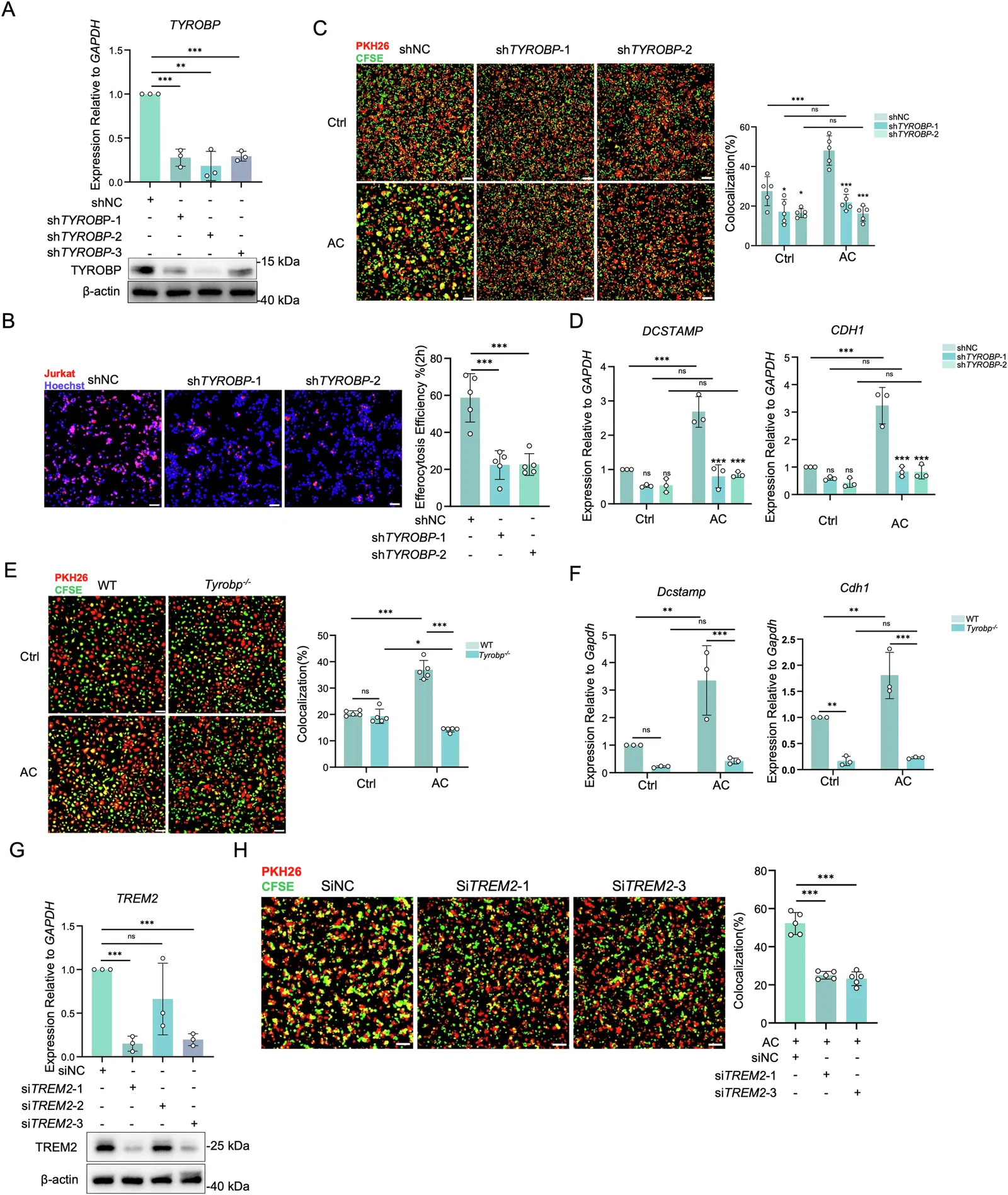
TYROBP affects apoptotic cell-mediated macrophage fusion. **A** Quantitative PCR and Western blot analysis of the mRNA and protein knockdown levels of TYROBP in THP-1-derived M0 (*n* = 3 per group). **B** PKH26-labeled apoptotic Jurkat were co-cultured with or without *TYROBP* knockdown in THP-1-derived M0 (Hoechst) (*n* = 5 per group). Scale bar, 50 μm. **C** THP-1-derived M0(Ctrl) were labeled with CFSE and PKH26 and fusion was induced by exposure to AC in control and *TYROBP* knockdown groups. Macrophage fusion is represented by colocalization of the red and green fluorescent labels (yellow) (*n* = 5 per group). Scale bar, 100 μm. **D** Quantitative PCR analysis of *DCSTAMP* and *CDH1*. (*n* = 3 per group). **E** BMDMs from WT and *Tyrobp*^−/−^ mice were labeled with CFSE and PKH26 and fusion was induced by exposure to AC. Macrophage fusion is represented by colocalization of the red and green fluorescent labels (yellow) (*n* = 5 per group). Scale bar, 100 μm. **F** Quantitative PCR analysis of *Dcstamp* and *Cdh1* (*n* = 3 per group). **G** Quantitative PCR and Western blot analysis of the mRNA and protein knockdown levels of TREM2 in THP-1-derived M0 (*n* = 3 per group). **H** THP-1-derived M0 with or without *TREM2* knockdown were labeled with CFSE and PKH26 and fusion was induced by exposure to AC. Macrophage fusion is represented by colocalization of the red and green fluorescent labels (yellow) (*n* = 5 per group). Scale bar, 100 μm. Data are representative of three independent experiments. Mean ± SD, *p < 0.05, **p < 0.01, ***p < 0.001.

At the receptor-signaling stage, *TYROBP* knockdown significantly reduced the efferocytic capacity of THP-1-derived M0 macrophages (Fig. 7A, B) and suppressed apoptotic cell-induced macrophage fusion and DCSTAMP/CDH1 expression (Fig. 7C, D). Consistent results were obtained in *Tyrobp*^−/−^ BMDMs (Fig. 7E, F). Knockdown of *TREM2*, a broad damage-associated lipid sensor coupled to TYROBP [54, 55], likewise reduced macrophage fusion (Fig. 7G, H). These findings support a contribution of TREM2-TYROBP signaling to efficient fusion but do not establish this pathway as the exclusive mediator of apoptotic-cell recognition or engulfment.

**Fig. 8:**
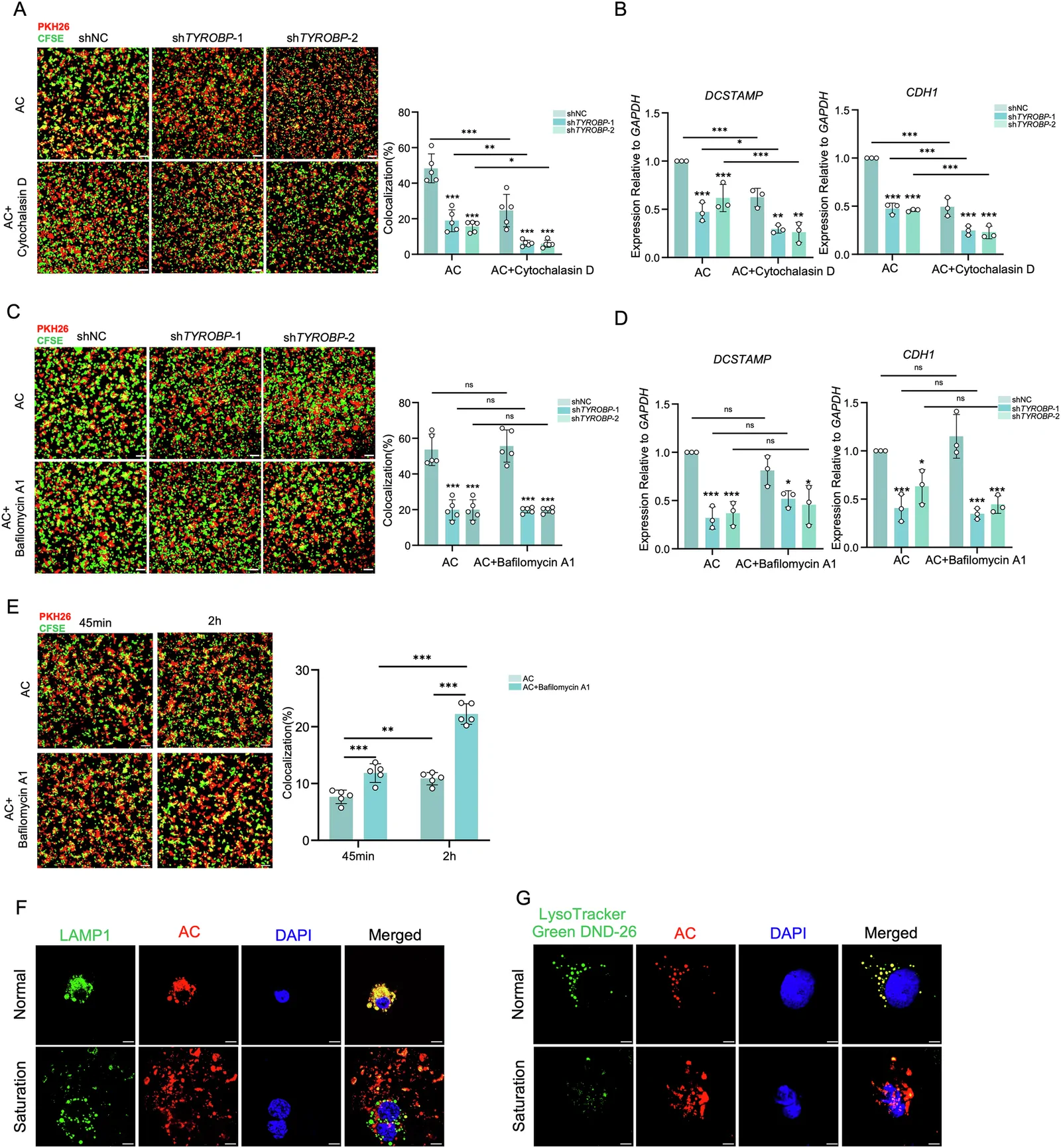
Apoptotic cell overload-induced macrophage fusion involves efferocytosis-associated signaling and impaired apoptotic-cell degradation. THP-1-derived M0 were labeled with CFSE and PKH26 and fusion was induced in control and *TYROBP* knockdown groups under AC or AC plus 5 μM Cytochalasin D (**A**) or 1 μM Bafilomycin A1 (**C**) treatment. Macrophage fusion is represented by colocalization of the red and green fluorescent labels (yellow) (*n* = 5 per group). Scale bar, 100 μm. **B**, **D** Quantitative PCR analysis of *DCSTAMP* and *CDH1* (*n* = 3 per group). **E** Apoptotic cells were co-cultured with THP-1-derived M0 macrophages at a 1:1 ratio to assess the effects of incubation time and Bafilomycin A1 on macrophage fusion. (*n* = 5 per group). Scale bar, 100 μm. **F**, **G** Immunofluorescence analysis of the colocalization between lysosomes and efferosomes in MGCs formed under normal efferocytosis conditions (1:1, 4 h) and apoptotic cell overload conditions (1:5, 4 h). Scale bar, 10 μm. Small-molecule compound used in this study are listed in Supplementary Table S7. Mean ± SD, *p < 0.05, **p < 0.01, ***p < 0.001.

Cytochalasin D (CytoD) significantly reduced macrophage fusion and DCSTAMP/CDH1 transcription (Fig. 8A, B). *TYROBP* knockdown independently reduced these readouts and produced a further reduction when combined with CytoD; two-way ANOVA showed a significant interaction (37.71% of total variation, *p* < 0.0001). Similar results were obtained in *Tyrobp*^−/−^ BMDMs (Supplementary Fig. S1B, C). Because CytoD broadly disrupts actin-dependent processes required for both phagocytic internalization and cell-cell fusion, these data support a requirement for actin-dependent cytoskeletal remodeling but do not distinguish impaired apoptotic-cell uptake from direct inhibition of the fusion machinery. They also indicate that receptor signaling alone is insufficient when downstream actin-dependent remodeling is blocked.

At the post-engulfment degradation stage, under overload conditions (1:5), inhibition of lysosomal acidification with bafilomycin A1 (Baf A1) had no significant effect on fusion, whereas *TYROBP* knockdown on this background still reduced fusion and *DCSTAMP*/*CDH1* expression (Fig. 8C, D). Consistent findings were obtained in *Tyrobp*^−/−^ BMDMs (Supplementary Fig. S1D, E). These results suggest that under apoptotic-cell overload conditions, the post-engulfment degradation step may already be saturated or functionally impaired, such that further inhibition of lysosomal acidification cannot additionally enhance fusion. In contrast, under the non-overload efferocytic condition (1:1), blocking lysosomal acidification and thereby impairing degradation significantly promoted, rather than inhibited, macrophage fusion (Fig. 8E), indicating that post-engulfment degradation blockade is a key factor driving macrophages away from complete efferocytosis and toward fusion differentiation.

To further test this notion at the organelle level, we compared the colocalization of lysosomes and efferosomes in MGCs formed under apoptotic-cell overload conditions (macrophage:apoptotic cell = 1:5, 4 h) with that observed under non-overload efferocytosis conditions (1:1, 4 h). Relative to the clear lysosome-efferosome colocalization seen under non-overload efferocytosis, MGCs formed under overload conditions displayed significantly reduced lysosome-efferosome colocalization (Fig. 8F, G), supporting the idea that the post-engulfment process of lysosome docking/fusion and degradation are impaired under overload conditions.

### Single-cell analysis identifies therapy-associated fusion-like myeloid/macrophage populations in colorectal cancer

**Fig. 9:**
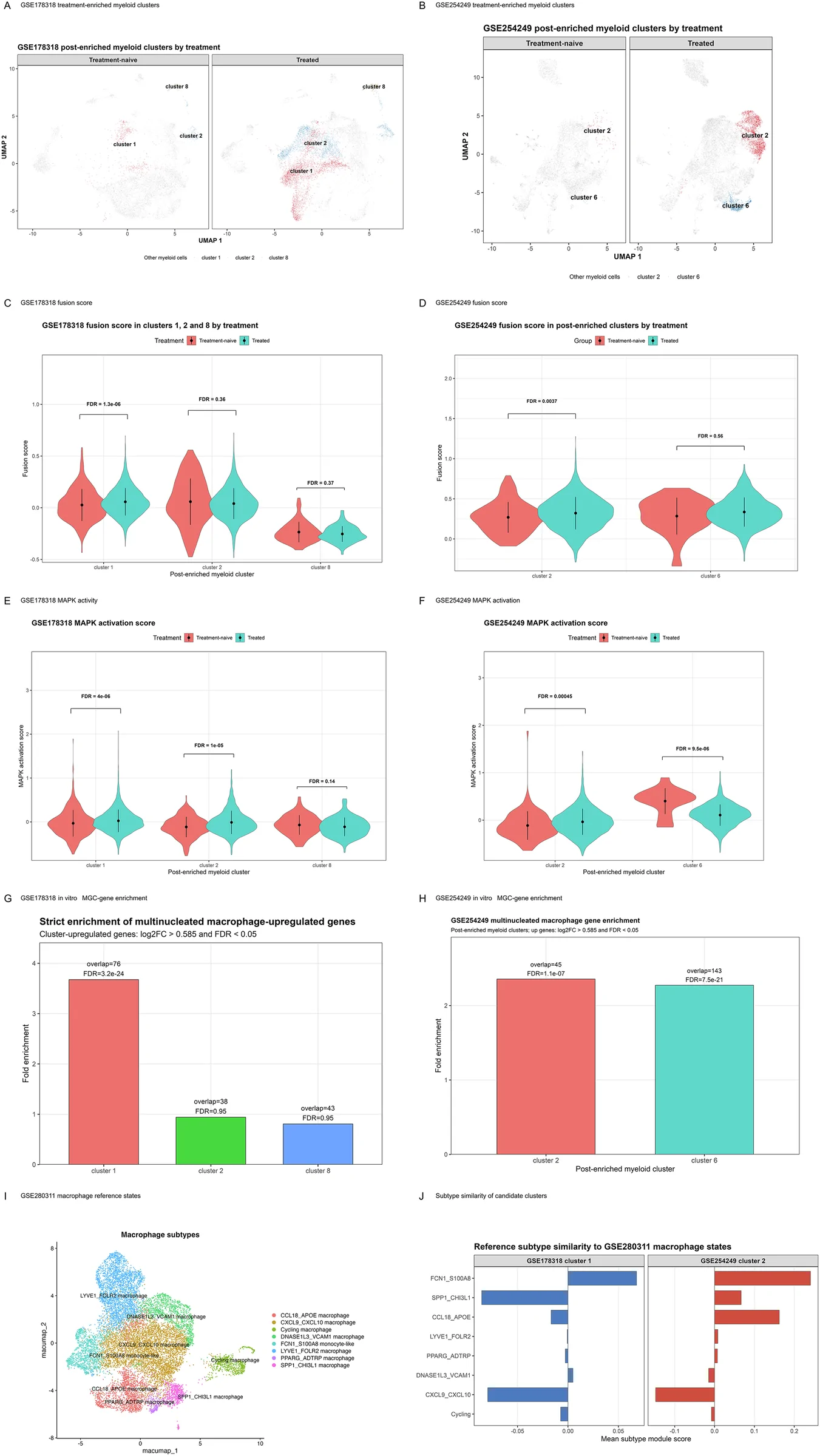
Single-cell identification of therapy-associated fusion-like myeloid/macrophage populations in colorectal cancer. UMAP visualization of treatment-enriched myeloid clusters in GSE178318 (**A**) and GSE254249 (**B**). Treatment-naive cells are shown on the left and treated cells on the right. Candidate post-treatment enriched clusters are highlighted, while other myeloid cells are shown in gray. Fusion score comparison between treatment-naive and treated cells in candidate clusters from GSE178318 (**C**) and GSE254249 (**D**). Black points and bars indicate mean ± SD. MAPK activation score comparison between treatment-naive and treated cells in candidate clusters from GSE178318 (**E**) and GSE254249 (**F**). Black points and bars indicate mean ± SD. Hypergeometric enrichment analysis comparing cluster-upregulated genes from GSE178318 clusters (**G**) and GSE254249 clusters (**H**) with genes upregulated in our in vitro MGC RNA-seq model. Fold enrichment and FDR values are shown for each candidate cluster. **I** UMAP annotation of macrophage subtypes from the GSE280311 colorectal cancer macrophage reference dataset, showing eight annotated macrophage states. **J** Reference mapping of candidate fusion-like populations to GSE280311 macrophage states. GSE178318 cluster 1 and GSE254249 cluster 2 showed the closest similarity to the FCN1/S100A8 monocyte-like macrophage state.

To determine whether MGC-associated macrophage states also emerge in colorectal cancer after neoadjuvant therapy, we analyzed two independent single-cell RNA-seq datasets containing paired or treatment-stratified colorectal cancer samples, GSE178318 and GSE254249. Myeloid cells were reclustered and treatment-enriched populations were identified based on post-treatment cell proportion and cluster abundance. In GSE178318, cluster 1 was enriched after treatment and expressed macrophage-related markers including *RETN*, *AREG*, *CD163*, *THBS1*, *TYROBP*, and *FCN1*, whereas other treatment-enriched clusters were more consistent with proliferating myeloid cells or DC-like cells. In GSE254249, cluster 2 was similarly enriched after treatment and expressed inflammatory and tissue-remodeling myeloid markers, including *MMP9*, *CXCL5*, *MT1G*, *SLAMF9*, *SPP1*, and *IL4I1* (Fig. 9A, B).

Although *DCSTAMP/TM7SF4* expression was detectable and relatively enriched within monocyte/macrophage compartments across the EMTAB8107 and GSE166555 colorectal cancer single-cell datasets (Supplementary Fig. S1H), only a small subset of individual monocytes/macrophages showed detectable expression. Given that *DCSTAMP/TM7SF4* expression may be transient and susceptible to dropout in scRNA-seq, we therefore evaluated a broader fusion-associated macrophage program comprising *DCSTAMP*, *OCSTAMP*, *ATP6V0D2*, *CDH1*, *CD9*, *CD44*, *SIRPA*, *ADAM8*, *ADAM9*, *ADAM12*, *MMP9*, *MMP14*, *ITGAM*, *ITGB2*, *ITGAX*, *TYROBP*, and *TREM2*. This fusion score was significantly increased after treatment in GSE178318 cluster 1 and GSE254249 cluster 2 (Fig. 9C, D).

Both populations also showed increased MAPK activation scores after treatment (Fig. 9E, F), and their cluster-upregulated genes were significantly enriched for genes induced in our in vitro MGC RNA-seq model (Fig. 9G, H).

Finally, comparison with GSE280311 colorectal cancer macrophage reference states showed that both populations most closely resembled *FCN1*/*S100A8* monocyte-like macrophages rather than mature *SPP1*/*CHI3L1* TAMs, suggesting that they may derive from recently recruited inflammatory monocyte-like cells that acquire partial MGC-associated remodeling features under therapy-induced apoptotic and tissue stress (Fig. 9I, J).

Together, these results indicate that neoadjuvant therapy is associated with the emergence of an *ADAM9*-*CD44*-*SIRPA*-*TYROBP* inflammatory myeloid/macrophage population with fusion-like and MGC-associated transcriptional features in colorectal cancer.

### Lysosomal function is reduced after apoptotic cell overload-induced MGC formation

**Fig. 10:**
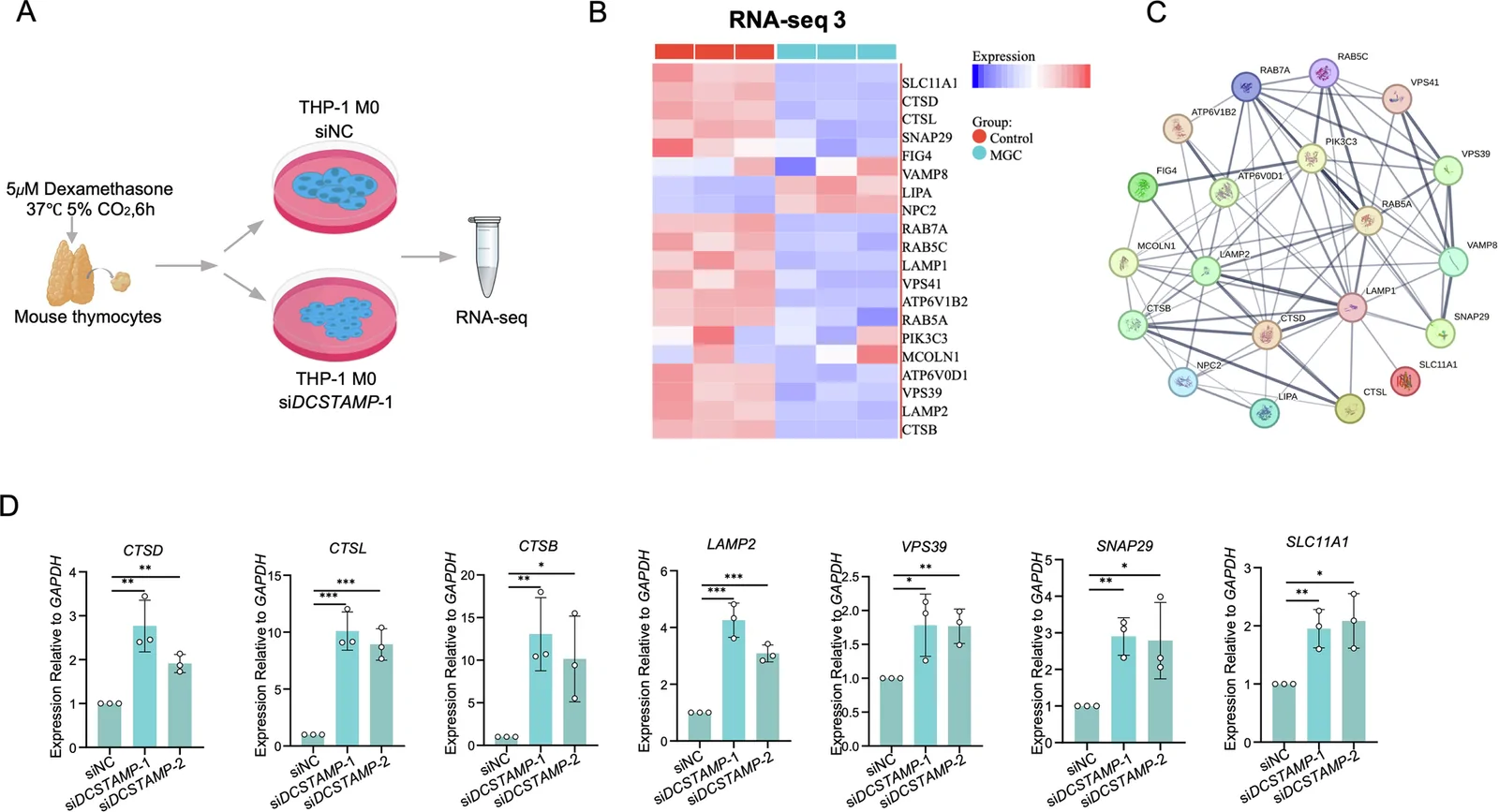
Apoptotic cell overload drives macrophage fusion through lysosomal decompensation and metabolic dysfunction. **A** Experimental design for RNA-seq comparing fused macrophages (MGC-enriched, WT) and non-fusing macrophages (*DCSTAMP* knockdown) following apoptotic cell overload (*n* = 3 per group). **B** Heatmap showing changes of lysosomal function-related genes in MGCs compared with *DCSTAMP*-deficient macrophages under apoptotic cell overload conditions. **C** Lysosomal gene network. **D** Quantitative PCR analysis of selected lysosomal genes in MGCs compared with non-fusing macrophages (*n* = 3 per group). Mean ± SD, *p < 0.05, **p < 0.01, ***p < 0.001.

Because the RNA-seq samples generated from the above apoptotic cell co-culture model may contain both macrophages that tend toward an immunoregulatory and pro-resolving state after efferocytosis and macrophages that have already undergone fusion into MGCs, we further compared, by RNA-seq, fusion-competent macrophages generated after apoptotic-cell overload co-culture (WT: containing a higher proportion of MGCs) with macrophages that failed to undergo fusion because of *DCSTAMP* knockdown (*DCSTAMP* KD) (Fig. 10A), in order to define the transcriptional features associated with the post-fusion MGC state.

Analysis of gene sets related to metabolism and organelle function showed that, compared with the *DCSTAMP* KD control group, the MGC group exhibited downregulation of multiple lysosome-related genes (Fig. 10B), including genes involved in lysosomal/phagosomal membrane transport and acidification (*SLC11A1*, *ATP6V1B2*, *LAMP1*), membrane trafficking and maturation (*SNAP29*, *RAB7A*, *RAB5A/C*, *VPS41*), and proteolysis (*CTSB*). These findings suggest that, under conditions of apoptotic-cell overload, MGC formation is accompanied by reduced lysosomal function.

We further constructed and analyzed a lysosome-related gene network (Fig. 10C), in which seven genes were significantly reduced in the WT (MGC-enriched) group (log_2_FC < −0.585, FDR < 0.05), including the cathepsins *CTSD*/*CTSL*/*CTSB*, the phagosome-lysosome fusion/maturation factors *LAMP2* and *VPS39*, *SNAP29*, which participates in membrane fusion during LC3-associated phagocytosis (LAP), and the divalent metal ion transporter *SLC11A1* on the phagosomal/lysosomal membrane, which regulates phagosomal microenvironment and metal ion homeostasis. qPCR validation confirmed that all of these genes were significantly downregulated in the WT (MGC-enriched) group (Fig. 10D). Together, these results suggest that apoptotic cell-induced MGCs adopt a functional state characterized by reduced processing of engulfed material.

### Apoptotic cell-induced MGCs exert anti-tumor effects

Previous studies have shown that nanoparticle-induced MGCs can exert anti-tumor effects [22]. However, whether MGCs generated after NAC merely represent a bystander phenomenon associated with increased post-treatment apoptotic burden or instead actively remodel the immune microenvironment and influence tumor progression, remains unclear.

**Fig. 11:**
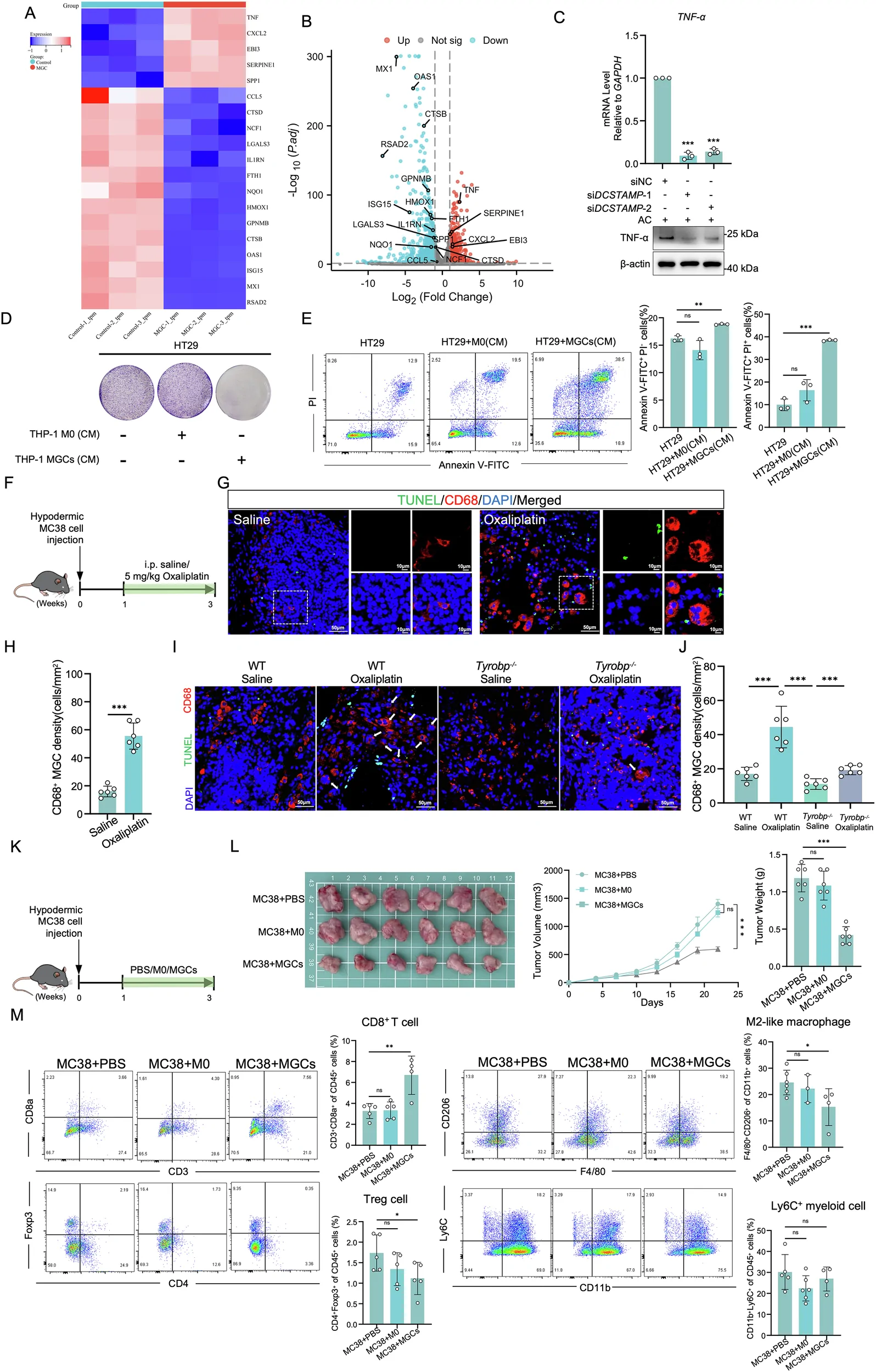
Apoptotic cell-induced MGCs exhibit antitumor effects and promote tumor immunity. **A** Heatmap of 19 selected differentially expressed genes with |log_2_FC| > 0.585, FDR < 0.05, and mean TPM > 100 from RNA-seq analysis of THP-1-derived M0 exposed to AC with or without *DCSTAMP* knockdown (*n* = 3 per group). **B** Volcano plot showing genes with significant expression changes (*n* = 3 per group). **C** Quantitative PCR and Western blot analysis of the levels of TNF-α and β-actin (loading control) in THP-1-derived M0 in groups with or without *DCSTAMP* knockdown. **D** Proliferation of HT29 cells following co-culture with conditioned medium (CM) from THP-1-derived M0 or MGCs. Data are shown as representative pictures. **E** FACS analysis of apoptosis in HT29 cells co-cultured with CM from THP-1 M0 macrophages or MGCs (*n* = 3 per group). **F** Schematic of the oxaliplatin-treated MC38 tumor experiment in wild-type and *Tyrobp*^−/−^ mice. **G**, **I** Representative immunofluorescence images of CD68^+^ MGCs and apoptosis in tumors from indicated groups (*n* = 6 per group). Scale bars: 50 μm and 10 μm. **H**, **J** Quantification of CD68^+^ MGC density for the corresponding panels (*n* = 6 per group). **K** Schematic representation of the experiments in (**L**) and (**M**). **L** Schematic representation of tumor size (left), growth curve analysis (medium), and comparative statistical analysis of tumor weight (right) in designated experimental groups. (*n* = 6 per group). **M** FACS analysis of CD206⁺ macrophages, Ly6C^+^ myeloid cells, Treg cell and CD8^+^ T cell proportions in designated experimental groups. Data are presented as representative plots and summary graphs of cell proportions. Mean ± SD, *p < 0.05, **p < 0.01, ***p < 0.001.

To identify secreted factors that might mediate the effects of MGCs on tumors, we performed RNA-seq analysis comparing efferocytosis-induced MGCs with macrophages in which fusion was blocked by *DCSTAMP* knockdown. Because paracrine signals are more likely to mediate the distal effects of MGCs on tumor cells and the immune microenvironment, we focused on highly expressed candidate secreted/soluble factors to reduce false positives caused by low-abundance transcripts (FDR < 0.05, |Log_2_(Fold Change)| >0.585, Control_TPM_mean >100 or MGC_TPM_mean >100) (Fig. 11A and Supplementary Table S6). The results showed that *TNF-α*, *CXCL2*, *EBI3*, *SERPINE1*, and *SPP1* were significantly upregulated in the MGC group, whereas *CCL5*, *CTSD*, *NCF1*, *LGALS3*, *IL1RN*, *FTH1*, *NQO1*, *HMOX1*, *GPNMB*, *CTSB*, and multiple IFN/ISG-related genes (*OAS1*, *ISG15*, *MX1*, *RSAD2*) were significantly downregulated (Fig. 11B). These findings suggest that the secretory profile of MGCs includes expression changes consistent with both potential pro-inflammatory anti-tumor activity and reduced immune-suppressive programs. Among these factors, TNF-α showed the greatest degree of upregulation in the RNA-seq analysis and was further validated at both the protein and mRNA levels (Fig. 11C).

Functionally, conditioned medium from MGCs significantly inhibited colorectal cancer-cell growth and promoted apoptosis compared with conditioned medium from M0 macrophages (Fig. 11D, E and Supplementary Fig. S1F, G). We next established a subcutaneous tumor model in mice and administered chemotherapy intraperitoneally to mimic the treatment conditions in patients. Immunofluorescence staining of tumor tissues confirmed the presence of MGCs (Fig. 11F–H). Notably, MGC formation was reduced in *Tyrobp*^−/−^ mice (Fig. 11I, J), indicating that *TYROBP* plays an essential role in promoting MGC formation in vivo.

Furthermore, in a subcutaneous syngeneic mouse model of colorectal cancer, we compared the effects of intratumoral injection of MGCs with those of M0 macrophages and found that MGCs significantly suppressed tumor growth (Fig. 11K, L). Given that MGCs may act through immunomodulatory mechanisms, we further analyzed immune infiltration within the TME. MGC treatment reduced the proportions of CD206⁺ macrophages and regulatory T cells while increasing the proportion of CD8^+^ T cells (Fig. 11M and Supplementary Fig. S2), suggesting that their anti-tumor effects are closely associated with remodeling of the immune microenvironment.

## Discussion

**Fig. 12:**
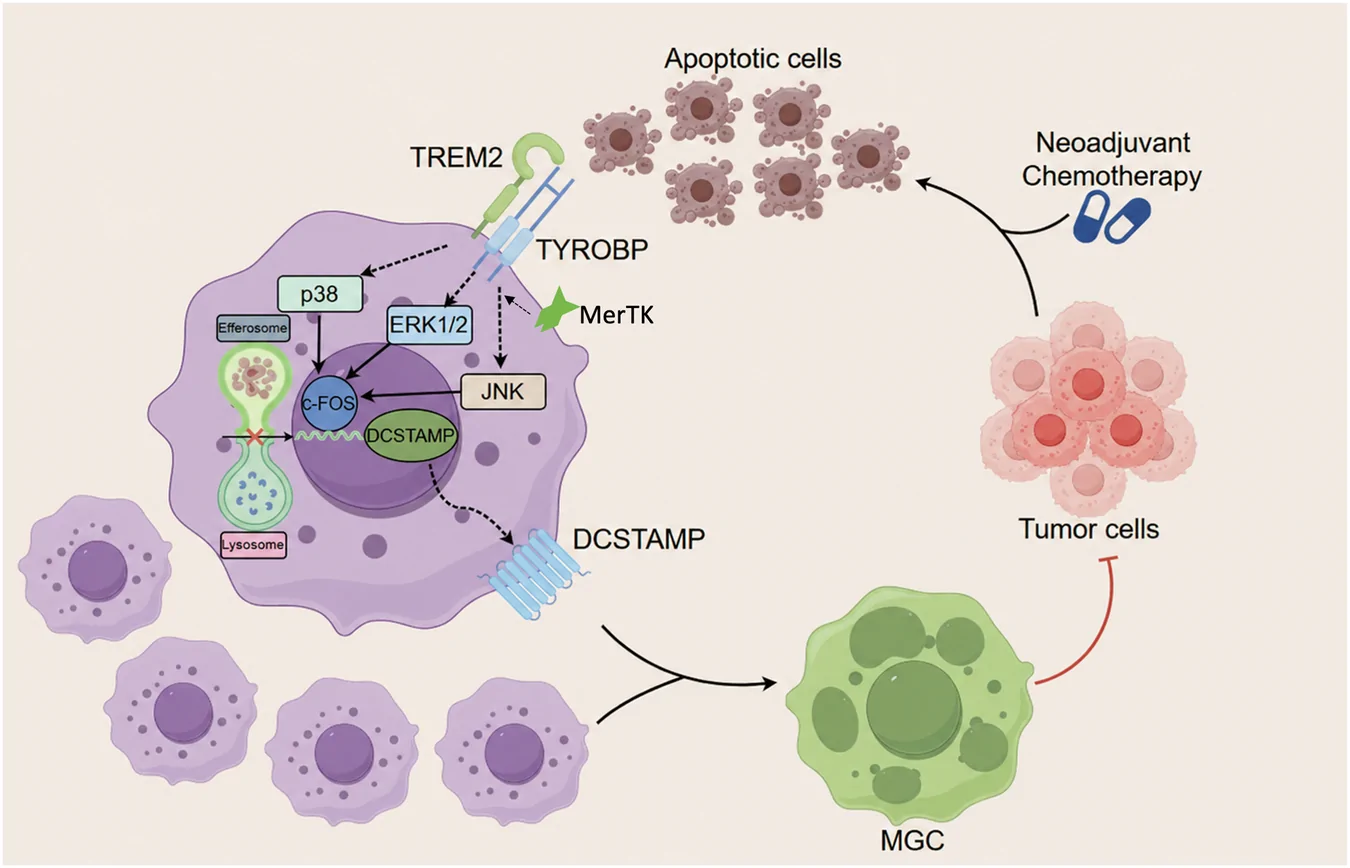
Working model of apoptotic cell overload-induced MGC formation after NAC. Neoadjuvant chemotherapy increases apoptotic-cell burden within the tumor microenvironment. Multiple receptor inputs, including TREM2-TYROBP and MerTK, may act in parallel or converge on MAPK signaling, promoting c-FOS nuclear translocation and *DCSTAMP* transcription. Together with actin-dependent remodeling and impaired lysosomal processing, this signaling state promotes macrophage fusion into MGCs. The resulting MGCs may suppress tumor growth through altered secretory programs and immune-microenvironment remodeling.

In summary, we found that the increased apoptotic burden induced by NAC promotes macrophage fusion through efferocytosis-associated receptor signaling, actin-dependent remodeling, and activation of the MAPK-c-FOS-DCSTAMP program (Fig. 12). TREM2-TYROBP is an important input into this fusion response, while the similar effect of MerTK inhibition indicates that multiple receptor pathways may act in parallel or converge on MAPK signaling. This process is independent of the classical IL-4-STAT6 and RANKL-NFATc1 fusion programs. Functionally, conditioned medium from MGCs suppressed colorectal cancer-cell proliferation and promoted apoptosis, while MGC administration in vivo inhibited tumor growth, reduced CD206^+^ macrophages and regulatory T cells, and increased CD8^+^ T-cell infiltration.

Previous studies in patients with head and neck squamous cell carcinoma (HNSCC) have shown that chemotherapy can induce abundant MGC formation, with TREM2^High^ TAMs and MGCs accumulating in keratin-containing tumor niches, leading to the enrichment of TREM2^High^ TAMs and their subsequent fusion into anti-tumor MGCs [56]. This finding is highly consistent with the pathway observed in our NAC-related colorectal cancer model—namely, increased apoptotic burden, TREM2 axis-driven fusion, and the generation of immunoregulatory MGCs—suggesting that MGCs may represent a plastic TME response under specific therapeutic stress conditions.

In addition, biomaterial implantation can trigger host foreign-body reactions and lead to the formation of foreign body giant cells (FBGCs), which in turn suppress tumor progression [22]. Increasing evidence also indicates that FBGCs exert beneficial functions in modulating the surrounding immune environment [57, 58] as well as in phagocytosis and related processes [59, 60]. These observations are consistent with the beneficial effects of MGCs identified in our study. Because MGCs arise through distinct mechanisms in different tissues and disease contexts, their biological functions may also differ substantially. Previous studies have further shown that efferocytosis of different types of apoptotic cells can confer distinct functional outcomes on macrophages [61]. This raises several important questions: do MGCs formed in different tissues or disease settings represent reproducible molecular subtypes? Do different classes of apoptotic cells (tumor cells, immune cells, or stromal cells) induce MGCs that affect tumors through distinct paracrine axes, such as IL-27, TGF-β, or IL-10? These issues will require systematic comparison in patient samples and across multiple models.

Previous studies have shown that macrophage efferocytosis of apoptotic tumor cells often shifts macrophages toward an immunoregulatory, tumor-supportive state, thereby promoting immune escape and tumor progression [62]. In contrast, we found that macrophage efferocytosis of apoptotic tumor cells can promote MGC formation and suppress tumors. Our data suggest that, under a specific therapy-induced high-apoptotic-burden context, efferocytosis can push macrophages into a fusion-MGC trajectory in the setting of MAPK activation and reduced lysosomal function, thereby producing anti-tumor effects through immune remodeling. We speculate that this divergence may depend on the timing of efferocytosis, the intensity of apoptotic burden, and downstream signaling bias: TYROBP may facilitate both efferocytosis and fusion during cytoskeletal rearrangement and phagocytic cup formation, whereas activation of downstream MAPK signaling together with overload-associated lysosomal dysfunction may lock macrophages into a fusion fate before they enter the pro-resolving and immunoregulatory program, thereby altering their subsequent polarization trajectory.

Our findings suggest that apoptotic-cell overload-induced macrophage fusion is not exclusively controlled by TREM2-TYROBP. Apoptotic-cell recognition involves a partially redundant network of efferocytic receptors, several of which can activate MAPK signaling. In our system, MerTK inhibition reduced MAPK phosphorylation, *DCSTAMP* expression, and macrophage fusion, identifying MerTK as an additional upstream input into the MAPK-DCSTAMP fusion program. This is particularly relevant because TREM2 is a broad sensor of damage-associated lipids rather than an apoptotic-cell-specific receptor, and its contribution to efferocytosis appears to be context dependent. For example, in an Aβ-associated model, TREM2 primarily supported microglial survival and plaque-associated responses, whereas its loss did not directly impair apoptotic-cell engulfment [63]. Thus, although disruption of TREM2-TYROBP markedly suppressed macrophage fusion in our model, the similar effects of MerTK inhibition indicate that multiple receptor pathways may act in parallel or converge on MAPK signaling to promote DCSTAMP induction and fusion. Whether efficient MGC formation requires coordinated engagement of TREM2-TYROBP and MerTK, or instead reflects a broader signaling network activated by apoptotic-cell overload and additional cues within the tumor microenvironment, remains to be determined through combinatorial genetic and pharmacological studies.

The Cyto D results should be interpreted cautiously, as Cyto D may directly inhibit actin-dependent fusion and therefore cannot specifically demonstrate the effect of apoptotic-cell engulfment on MGC formation. Alternative approaches, such as masking phosphatidylserine with Annexin V, blocking efferocytic receptors such as TIM-4 or MERTK, or inhibiting lysosomal degradation with bafilomycin A1, may also affect recognition, downstream signaling, or broader cellular functions. Future studies using more specific strategies are needed to clarify how different stages of efferocytosis regulate macrophage fusion.

Although our data support a role for efferocytosis-associated receptor signaling and MAPK activation in apoptotic-cell overload-induced macrophage fusion, contributions from apoptotic-cell-derived soluble factors cannot be excluded. These may include cytokines, lipids, damage-associated molecules, or extracellular vesicles, particularly in the presence of secondary necrosis. Because conditioned-medium and fractionation experiments were beyond the scope of this study, our findings do not establish direct contact or engulfment as the sole driver of MGC formation. Further studies are needed to determine how soluble signals cooperate with efferocytic receptor pathways.

The preferential enrichment of fusion-associated features in FCN1/S100A8-like macrophages may reflect the greater migratory and developmental plasticity of newly recruited monocyte-derived cells. Following neoadjuvant therapy, sustained apoptotic-cell burden and tissue stress may divert these transitional cells toward a TYROBP-MAPK/c-FOS-DCSTAMP-associated, fusion-competent state before they fully acquire more differentiated TAM programs characterized by SPP1, APOE, PPARG, or related tissue-adaptive features. This model does not exclude the involvement of differentiated TAMs, which may participate as relatively mature recipient or founder cells that fuse with more motile monocyte-like partners. Therapy-associated MGC formation may therefore involve heterotypic fusion between macrophages at distinct stages of differentiation rather than fusion within a single predefined macrophage subset. Lineage tracing and spatially resolved analyses will be required to determine the relative contributions of recruited monocytes and established TAM populations.

It is well established that the TREM2-TYROBP axis can promote TAM differentiation and exert pro-tumor functions in multiple cancers [64, 65], and the TREM2 inhibitor PY-314 has also been proposed as an anti-cancer strategy by suppressing TAM differentiation [66]. Our findings suggest that during the window of markedly increased apoptotic burden induced by NAC, TREM2 signaling does not necessarily drive macrophages toward a pro-tumor TAM fate; instead, it may be redirected toward a fusion-MGC pathway with anti-tumor consequences. This implies that anti-cancer therapeutic strategies should not be simplistically equated with “blocking TREM2” or “eliminating TREM2^+^ cells.” Rather, the key lies in precisely modulating the specific myeloid subsets and functional outputs reshaped by TREM2 signaling. In particular, in the neoadjuvant treatment setting, TREM2 inhibition may carry the potential risk of weakening beneficial MGC responses and should therefore be evaluated cautiously in the context of treatment timing and TME status.

Fusion is a process highly dependent on membrane/cytoskeletal remodeling and vesicular trafficking. Under extreme burden, lipid toxicity or lysosomal dysfunction may force cells to switch from an efficient degradative mode to an emergency strategy of load sequestration. Thus, apoptotic cell overload is not equivalent to enhanced efferocytosis, but may instead reflect failure of the efferocytosis-lysosomal adaptation program. The precise mechanisms linking lysosomal compensation failure to active downregulation of lysosome-related gene expression remain to be further elucidated.

In conclusion, we identify an NAC-associated mode of MGC formation driven by apoptotic-cell burden and convergent efferocytosis-associated receptor-MAPK-c-FOS-DCSTAMP signaling, and show that these MGCs can suppress tumor growth through tumor-microenvironment remodeling.

## Supplementary information

Supplementary Table

Supplementary Figure

Original Data

## Data Availability

Public single-cell RNA-sequencing datasets analyzed in this study are available under accession numbers GSE178318, GSE254249, GSE280311, E-MTAB-8107, and GSE166555. Other data supporting the findings are provided in the article, Supplementary Information, Supplementary Tables, and Original Data files.

## References

[1] Smith HG, Nilsson PJ, Shogan BD, Harji D, Gambacorta MA, Romano A, et al. Neoadjuvant treatment of colorectal cancer: comprehensive review. BJS Open. 2024;8:zrae038. 10.1093/bjsopen/zrae038. PMC11094476 38747103

[2] Nair KG, Kamath SD, Chowattukunnel N, Krishnamurthi SS. Preoperative strategies for locally advanced colon cancer. Curr Treat Options Oncol. 2024;25:376–88. 10.1007/s11864-024-01184-6. PMC10894759 38349502

[3] Shang B, Jia Y, Chen G, Wang Z. Ku80 correlates with neoadjuvant chemotherapy resistance in human lung adenocarcinoma, but reduces cisplatin/pemetrexed-induced apoptosis in A549 cells. Respir Res. 2017;18:56. 10.1186/s12931-017-0545-6. PMC5387337 28399858

[4] Wang H, Guo S, Kim SJ, Shao F, Ho JWK, Wong KU, et al. Cisplatin prevents breast cancer metastasis through blocking early EMT and retards cancer growth together with paclitaxel. Theranostics. 2021;11:2442–59. 10.7150/thno.46460. PMC7797698 33500735

[5] Kasi PM, Hidalgo M, Jafari MD, Yeo H, Lowenfeld L, Khan U, et al. Neoadjuvant botensilimab plus balstilimab response pattern in locally advanced mismatch repair proficient colorectal cancer. Oncogene. 2023;42:3252–9. 10.1038/s41388-023-02835-y. PMC10611560 37731056

[6] Lișcu HD, Antone-Iordache IL, Atasiei DI, Anghel IV, Ilie AT, Emamgholivand T, et al. The impact on survival of neoadjuvant treatment interruptions in locally advanced rectal cancer patients. J Pers Med. 2024;14:266. 10.3390/jpm14030266. PMC10971105 38541008

[7] Park SY, Chang HJ, Kim DY, Jung KH, Kim SY, Park JW, et al. Is step section necessary for determination of complete pathological response in rectal cancer patients treated with preoperative chemoradiotherapy? Histopathology. 2011;59:650–9. 10.1111/j.1365-2559.2011.03980.x. 22014046

[8] Viale G, Fusco N. Pathology after neoadjuvant treatment—how to assess residual disease. Breast. 2022;62:S25–8. 10.1016/j.breast.2021.11.009. PMC9097800 34810049

[9] Ju WT, Xia RH, Zhu DW, Dou SJ, Zhu GP, Dong MJ, et al. A pilot study of neoadjuvant combination of anti-PD-1 camrelizumab and VEGFR2 inhibitor apatinib for locally advanced resectable oral squamous cell carcinoma. Nat Commun. 2022;13:5378. 10.1038/s41467-022-33080-8. PMC9472189 36104359

[10] Travis WD, Dacic S, Wistuba I, Sholl L, Adusumilli P, Bubendorf L, et al. IASLC multidisciplinary recommendations for pathologic assessment of lung cancer resection specimens after neoadjuvant therapy. J Thorac Oncol. 2020;15:709–40. 10.1016/j.jtho.2020.01.005. PMC8173999 32004713

[11] Usui Y, Matsunuma R, Yamaguchi K, Hayami R, Muramatsu A, Suzuki M, et al. Pathological complete response to neoadjuvant chemotherapy in a patient with HER2-positive squamous cell carcinoma of the breast. Case Rep Oncol. 2021;14:1536–41. 10.1159/000519746. PMC8613576 34899248

[12] Inzani F, Arciuolo D, Angelico G, Santoro A, Travaglino A, D’Alessandris N, et al. Assessing post-treatment pathologic tumor response in female genital tract carcinomas: an update. Front Oncol. 2022;12:814989. 10.3389/fonc.2022.814989. PMC8866564 35223496

[13] Qu Y, Emoto K, Eguchi T, Aly RG, Zheng H, Chaft JE, et al. Pathologic assessment after neoadjuvant chemotherapy for NSCLC: importance and implications of distinguishing adenocarcinoma from squamous cell carcinoma. J Thorac Oncol. 2019;14:482–93. 10.1016/j.jtho.2018.11.017. PMC6382593 30503889

[14] Verschoor YL, van de Haar J, van den Berg JG, van Sandick JW, Kodach LL, van Dieren JM, et al. Neoadjuvant atezolizumab plus chemotherapy in gastric and gastroesophageal junction adenocarcinoma: the phase 2 PANDA trial. Nat Med. 2024;30:519–30. 10.1038/s41591-023-02758-x. PMC10878980 38191613

[15] Arandjelovic S, Ravichandran KS. Phagocytosis of apoptotic cells in homeostasis. Nat Immunol. 2015;16:907–17. 10.1038/ni.3253. PMC4826466 26287597

[16] Boada-Romero E, Martinez J, Heckmann BL, Green DR. The clearance of dead cells by efferocytosis. Nat Rev Mol Cell Biol. 2020;21:398–414. 10.1038/s41580-020-0232-1. PMC7392086 32251387

[17] Doran AC, Yurdagul A, Tabas I. Efferocytosis in health and disease. Nat Rev Immunol. 2020;20:254–67. 10.1038/s41577-019-0240-6. PMC7667664 31822793

[18] Ahmadzadeh K, Vanoppen M, Rose CD, Matthys P, Wouters CH. Multinucleated giant cells: current insights in phenotype, biological activities, and mechanism of formation. Front Cell Dev Biol. 2022;10:873226. 10.3389/fcell.2022.873226. PMC9035892 35478968

[19] Fontaine MAC, Westra MM, Bot I, Jin H, Franssen A, Bot M, et al. Low human and murine Mcl-1 expression leads to a pro-apoptotic plaque phenotype enriched in giant-cells. Sci Rep. 2019;9:14547. 10.1038/s41598-019-51020-3. PMC6787218 31601924

[20] Nagenborg J, Goossens P, Biessen EAL, Donners MMPC. Heterogeneity of atherosclerotic plaque macrophage origin, phenotype and functions: implications for treatment. Eur J Pharmacol. 2017;816:14–24. 10.1016/j.ejphar.2017.10.005. 28989084

[21] Qiao JH, Mishra V, Fishbein MC, Sinha SK, Rajavashisth TB. Multinucleated giant cells in atherosclerotic plaques of human carotid arteries: identification of osteoclast-like cells and their specific proteins in artery wall. Exp Mol Pathol. 2015;99:654–62. 10.1016/j.yexmp.2015.11.010. 26551087

[22] Chen S, Xing Z, Geng M, Zhao R, Yang X, Zhu X, et al. Macrophage fusion event as one prerequisite for inorganic nanoparticle-induced antitumor response. Sci Adv. 2023;9:eadd9871. 10.1126/sciadv.add9871. PMC10355827 37467339

[23] Nakahashi-Oda C, Fujiyama S, Nakazawa Y, Kanemaru K, Wang Y, Lyu W, et al. CD300a blockade enhances efferocytosis by infiltrating myeloid cells and ameliorates neuronal deficit after ischemic stroke. Sci Immunol. 2021;6:eabe7915. 10.1126/sciimmunol.abe7915. 34652960

[24] Zhou Y, Tada M, Cai Z, Andhey PS, Swain A, Miller KR, et al. Human early-onset dementia caused by DAP12 deficiency reveals a unique signature of dysregulated microglia. Nat Immunol. 2023;24:545–57. 10.1038/s41590-022-01403-y. PMC9992145 36658241

[25] Zhang M, Duan Y, Gan H, Jiang N, Qin L, Luo Y, et al. TYROBP serve as potential immune-related signature genes in the acute phase of intracerebral hemorrhage. Sci Rep. 2024;14:20158. 10.1038/s41598-024-71132-9. PMC11364555 39215129

[26] Helming L, Tomasello E, Kyriakides TR, Martinez FO, Takai T, Gordon S, et al. Essential role of DAP12 signaling in macrophage programming into a fusion-competent state. Sci Signal. 2008;1:ra11. 10.1126/scisignal.1159665. PMC2673960 18957693

[27] Takaki R, Watson SR, Lanier LL. DAP12: an adapter protein with dual functionality. Immunol Rev. 2006;214:118–29. 10.1111/j.1600-065X.2006.00466.x. 17100880

[28] Tomasello E, Vivier E. KARAP/DAP12/TYROBP: three names and a multiplicity of biological functions. Eur J Immunol. 2005;35:1670–7. 10.1002/eji.200425932. 15884055

[29] Colonna M. The biology of TREM receptors. Nat Rev Immunol. 2023;23:580–94. 10.1038/s41577-023-00837-1. PMC9904274 36750615

[30] Che LH, Liu JW, Huo JP, Luo R, Xu RM, He C, et al. A single-cell atlas of liver metastases of colorectal cancer reveals reprogramming of the tumor microenvironment in response to preoperative chemotherapy. Cell Discov. 2021;7:80. 10.1038/s41421-021-00312-y. PMC8421363 34489408

[31] Gao Q, Ling X, Liao L, Tang F, Jiang Y, Qin S, et al. Remodeling of T and endothelial cells during total neoadjuvant therapy in rectal cancer. Cancer Cell. 2025;43:2282–97.e9. 10.1016/j.ccell.2025.10.008. 41202810

[32] Oliveira MF, Romero JP, Chung M, Williams SR, Gottscho AD, Gupta A, et al. High-definition spatial transcriptomic profiling of immune cell populations in colorectal cancer. Nat Genet. 2025;57:1512–23. 10.1038/s41588-025-02193-3. PMC12165841 40473992

[33] Stuart T, Butler A, Hoffman P, Hafemeister C, Papalexi E, Mauck WM 3rd, et al. Comprehensive integration of single-cell data. Cell. 2019;177:1888–902.e21. 10.1016/j.cell.2019.05.031. PMC6687398 31178118

[34] Miyamoto T. STATs and macrophage fusion. JAKSTAT. 2013;2:e24777. 10.4161/jkst.24777. PMC3772113 24069561

[35] Dufrançais O, Mascarau R, Poincloux R, Maridonneau-Parini I, Raynaud-Messina B, Vérollet C. Cellular and molecular actors of myeloid cell fusion: podosomes and tunneling nanotubes call the tune. Cell Mol Life Sci. 2021;78:6087–104. 10.1007/s00018-021-03875-x. PMC8429379 34296319

[36] Tirosh I, Izar B, Prakadan SM, Wadsworth MH 2nd, Treacy D, Trombetta JJ, et al. Dissecting the multicellular ecosystem of metastatic melanoma by single-cell RNA-seq. Science. 2016;352:189–96. 10.1126/science.aad0501. PMC4944528 27124452

[37] O’Donnell A, Odrowaz Z, Sharrocks AD. Immediate-early gene activation by the MAPK pathways: what do and don’t we know? Biochem Soc Trans. 2012;40:58–66. 10.1042/bst20110636. 22260666

[38] Lake D, Corrêa SA, Müller J. Negative feedback regulation of the ERK1/2 MAPK pathway. Cell Mol Life Sci. 2016;73:4397–413. 10.1007/s00018-016-2297-8. PMC5075022 27342992

[39] Wagle MC, Kirouac D, Klijn C, Liu B, Mahajan S, Junttila M, et al. A transcriptional MAPK pathway activity score (MPAS) is a clinically relevant biomarker in multiple cancer types. NPJ Precis Oncol. 2018;2:7. 10.1038/s41698-018-0051-4. PMC5871852 29872725

[40] Benjamini Y, Drai D, Elmer G, Kafkafi N, Golani I. Controlling the false discovery rate in behavior genetics research. Behav Brain Res. 2001;125:279–84. 10.1016/s0166-4328(01)00297-2. 11682119

[41] Wickham H. ggplot2. Wiley Interdiscip Rev Comput Stat. 2011;3:180–5. 10.1002/wics.147.

[42] Yagi M, Miyamoto T, Sawatani Y, Iwamoto K, Hosogane N, Fujita N, et al. DC-STAMP is essential for cell-cell fusion in osteoclasts and foreign body giant cells. J Exp Med. 2005;202:345–51. 10.1084/jem.20050645. PMC2213087 16061724

[43] Lin J, Xu A, Jin J, Zhang M, Lou J, Qian C, et al. MerTK-mediated efferocytosis promotes immune tolerance and tumor progression in osteosarcoma through enhancing M2 polarization and PD-L1 expression. Oncoimmunology. 2022;11:2024941. 10.1080/2162402x.2021.2024941. PMC8757471 35036076

[44] Wei YT, Wang XR, Yan C, Huang F, Zhang Y, Liu X, et al. Thymosin α-1 reverses M2 polarization of tumor-associated macrophages during efferocytosis. Cancer Res. 2022;82:1991–2002. 10.1158/0008-5472.CAN-21-4260. 35364609

[45] Naidu S, Vijayan V, Santoso S, Kietzmann T, Immenschuh S. Inhibition and genetic deficiency of p38 MAPK up-regulates heme oxygenase-1 gene expression via Nrf2. J Immunol. 2009;182:7048–57. 10.4049/jimmunol.0900006. 19454702

[46] Yang Y, Kim SC, Yu T, Yi YS, Rhee MH, Sung GH, et al. Functional roles of p38 mitogen-activated protein kinase in macrophage-mediated inflammatory responses. Mediators Inflamm. 2014;2014:352371. 10.1155/2014/352371. PMC3977509 24771982

[47] Bian H, Li F, Wang W, Zhao Q, Gao S, Ma J, et al. MAPK/p38 regulation of cytoskeleton rearrangement accelerates induction of macrophage activation by TLR4, but not TLR3. Int J Mol Med. 2017;40:1495–503. 10.3892/ijmm.2017.3143. PMC5627867 28949380

[48] Kodama J, Kaito T. Osteoclast multinucleation: review of current literature. Int J Mol Sci. 2020;21:5685. 10.3390/ijms21165685. PMC7461040 32784443

[49] He Y, Staser K, Rhodes SD, Liu Y, Wu X, Park SJ, et al. Erk1 positively regulates osteoclast differentiation and bone resorptive activity. PLoS ONE. 2011;6:e24780. 10.1371/journal.pone.0024780. PMC3178550 21961044

[50] Haure-Mirande JV, Audrain M, Ehrlich ME, Gandy S. Microglial TYROBP/DAP12 in Alzheimer’s disease: transduction of physiological and pathological signals across TREM2. Mol Neurodegener. 2022;17:55. 10.1186/s13024-022-00552-w. PMC9404585 36002854

[51] Mehrotra P, Ravichandran KS. Drugging the efferocytosis process: concepts and opportunities. Nat Rev Drug Discov. 2022;21:601–20. 10.1038/s41573-022-00470-y. PMC9157040 35650427

[52] Nagesh R, Kiran Kumar KM, Naveen Kumar M, Patil RH, Sharma SC. Regulation of Jun and Fos AP-1 transcription factors by JNK MAPKs signaling cascade in areca nut extract treated KB cells. Biochem Biophys Rep. 2021;27:101090. 10.1016/j.bbrep.2021.101090. PMC8350022 34401529

[53] Shan J, Donelan W, Hayner JN, Zhang F, Dudenhausen EE, Kilberg MS. MAPK signaling triggers transcriptional induction of cFOS during amino acid limitation of HepG2 cells. Biochim Biophys Acta Mol Cell Res. 2015;1853:539–48. 10.1016/j.bbamcr.2014.12.013. PMC4315773 25523140

[54] Peng Q, Malhotra S, Torchia JA, Kerr WG, Coggeshall KM, Humphrey MB. TREM2- and DAP12-dependent activation of PI3K requires DAP10 and is inhibited by SHIP1. Sci Signal. 2010;3:ra38. 10.1126/scisignal.2000500. PMC2900152 20484116

[55] Yao H, Coppola K, Schweig JE, Crawford F, Mullan M, Paris D. Distinct signaling pathways regulate TREM2 phagocytic and NFκB antagonistic activities. Front Cell Neurosci. 2019;13:457. 10.3389/fncel.2019.00457. PMC6795686 31649511

[56] Gessain G, Anzali AA, Lerousseau M, Mulder K, Bied M, Auperin A, et al. TREM2-expressing multinucleated giant macrophages are a biomarker of good prognosis in head and neck squamous cell carcinoma. Cancer Discov. 2024;14:2352–66. 10.1158/2159-8290.Cd-24-0018. PMC11609822 39270324

[57] Barbeck M, Motta A, Migliaresi C, Sader R, Kirkpatrick CJ, Ghanaati S. Heterogeneity of biomaterial-induced multinucleated giant cells: possible importance for the regeneration process? J Biomed Mater Res A. 2016;104:413–8. 10.1002/jbm.a.35579. 26422451

[58] Miron RJ, Bosshardt DD. Multinucleated giant cells: good guys or bad guys? Tissue Eng Part B Rev. 2017;24:53–65. 10.1089/ten.teb.2017.0242. 28825357

[59] Klopfleisch R. Macrophage reaction against biomaterials in the mouse model—phenotypes, functions and markers. Acta Biomater. 2016;43:3–13. 10.1016/j.actbio.2016.07.003. 27395828

[60] Yang HC, Park HC, Quan H, Kim Y. Immunomodulation of biomaterials by controlling macrophage polarization. In: Noh I, editor. Biomimetic medical materials: from nanotechnology to 3D bioprinting. Singapore: Springer Singapore; 2018. pp. 197–206. 10.1007/978-981-13-0445-3_12. 30471034

[61] Liebold I, Al Jawazneh A, Casar C, Lanzloth C, Leyk S, Hamley M, et al. Apoptotic cell identity induces distinct functional responses to IL-4 in efferocytic macrophages. Science. 2024;384:eabo7027. 10.1126/science.abo7027. 38574142

[62] Cheng M, Chen S, Li K, Wang G, Xiong G, Ling R, et al. CD276-dependent efferocytosis by tumor-associated macrophages promotes immune evasion in bladder cancer. Nat Commun. 2024;15:2818. 10.1038/s41467-024-46735-5. PMC10985117 38561369

[63] Wang Y, Cella M, Mallinson K, Ulrich JD, Young KL, Robinette ML, et al. TREM2 lipid sensing sustains the microglial response in an Alzheimer’s disease model. Cell. 2015;160:1061–71. 10.1016/j.cell.2015.01.049. PMC4477963 25728668

[64] Yan Y, Bai S, Han H, Dai J, Niu L, Wang H, et al. Knockdown of trem2 promotes proinflammatory microglia and inhibits glioma progression via the JAK2/STAT3 and NF-κB pathways. Cell Commun Signal. 2024;22:272. 10.1186/s12964-024-01642-6. PMC11094905 38750472

[65] Binnewies M, Pollack JL, Rudolph J, Dash S, Abushawish M, Lee T, et al. Targeting TREM2 on tumor-associated macrophages enhances immunotherapy. Cell Rep. 2021;37:109844. 10.1016/j.celrep.2021.109844. 34686340

[66] Jahchan NS, Binnewies M, Pollack JL, Mehta R, Dash S, Tun C, et al. Abstract LB071: Tuning the tumor myeloid microenvironment (TME) by targeting TREM2+ tumor-associated macrophages to overcome resistance to immune checkpoint inhibitors. Cancer Res. 2021;81:LB071. 10.1158/1538-7445.AM2021-LB071.

