# Supplementary material for: Apoptotic cell overload drives multinucleated giant cell formation after neoadjuvant chemotherapy in colorectal cancer: molecular mechanisms and tumor-suppressive effects
Source: Cell Death Dis. 2026 Oct 5;17(1):907. doi: 10.1038/s41419-026-09320-w (PMC13694713; doi:10.1038/s41419-026-09320-w)
Supplement: Supplementary file 1 [file 41419_2026_9320_MOESM2_ESM.pdf]

Supplementary Figure S1

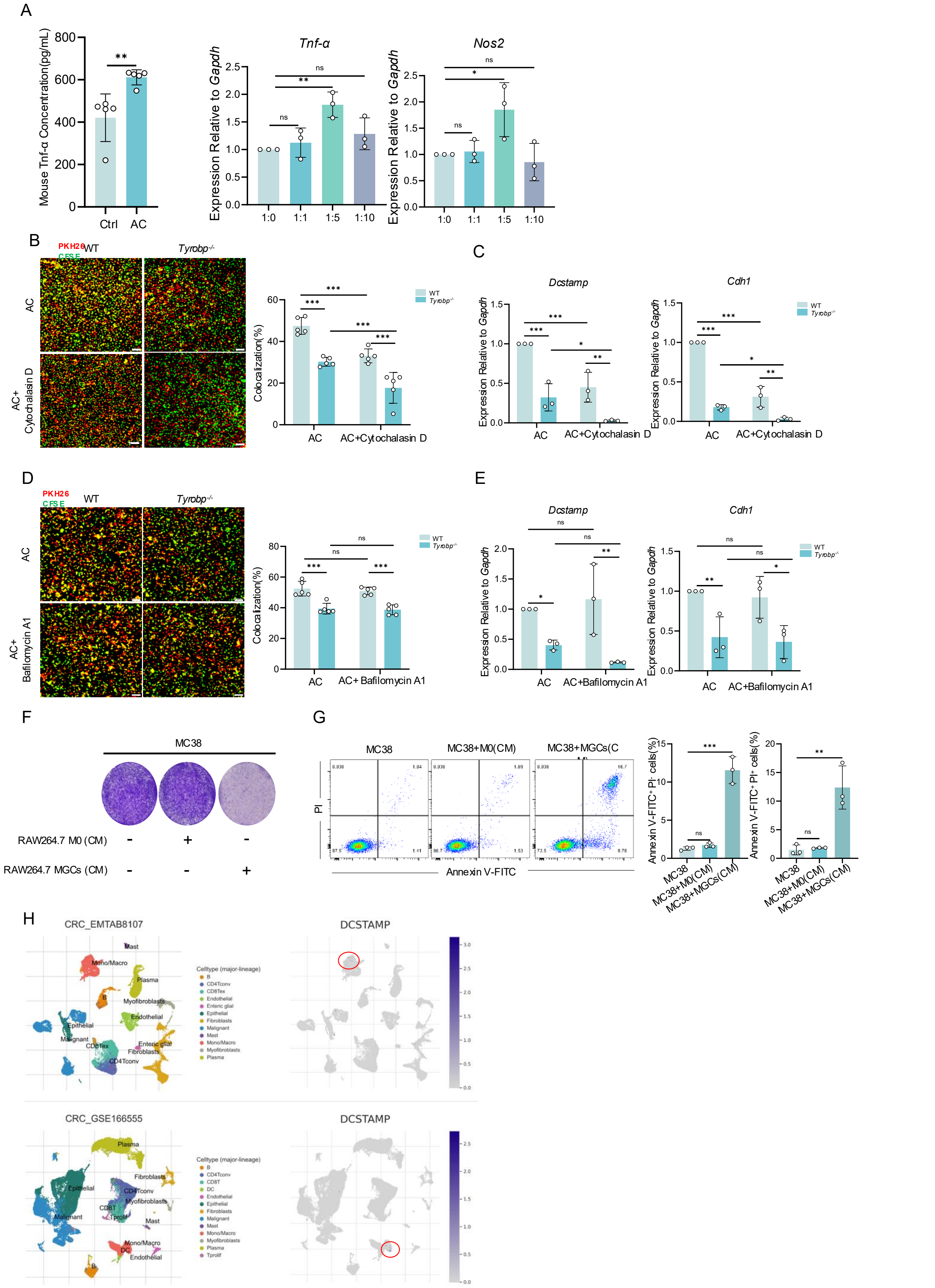

Suppl. Fig. S1. A. ELISA and quantitative PCR analysis of *Tnf-α*/*Nos2* in RAW264.7 with or without AC treatment (n= 5 per group). **B, D.** BMDM from WT or *Tyrobp-/-* mice were labeled with CFSE and PKH26 and fusion was induced with AC or AC and 5 μ M Cytochalasin D (**B**) or 1 μ M Bafilomycin A1 (D) treatment. Macrophage fusion is represented by colocalization of the red and green fluorescent labels (yellow) (n= 5 per group). Scale bar, 100 μm. **C, E.** Quantitative PCR analysis of *Dcstamp* and *Cdh1* (n= 3 per group). **F.** Proliferation of MC38 cells following co-culture with conditioned medium (CM) from RAW264.7 or MGCs. Data are shown as representative pictures. **G.** FACS analysis of apoptosis in MC38 cells co-cultured with CM from RAW264.7 M0 macrophages or MGCs (n= 3 per group). Small-molecule compounds used in this study are listed in Suppl. Table S7. H. UMAP visualization of major cell types (left) and DCSTAMP/TM7SF4 expression (right) in the EMTAB8107 (top) and GSE166555 (bottom) colorectal cancer single-cell RNA-seq datasets. *DCSTAMP* expression was relatively enriched in monocyte/macrophage compartments but was detectable in only a small subset of cells. Color intensity indicates normalized expression levels. Mean ± SD, *$P < 0.05$, **$P < 0.01$, ***$P < 0.001$.

Supplementary FigureS2

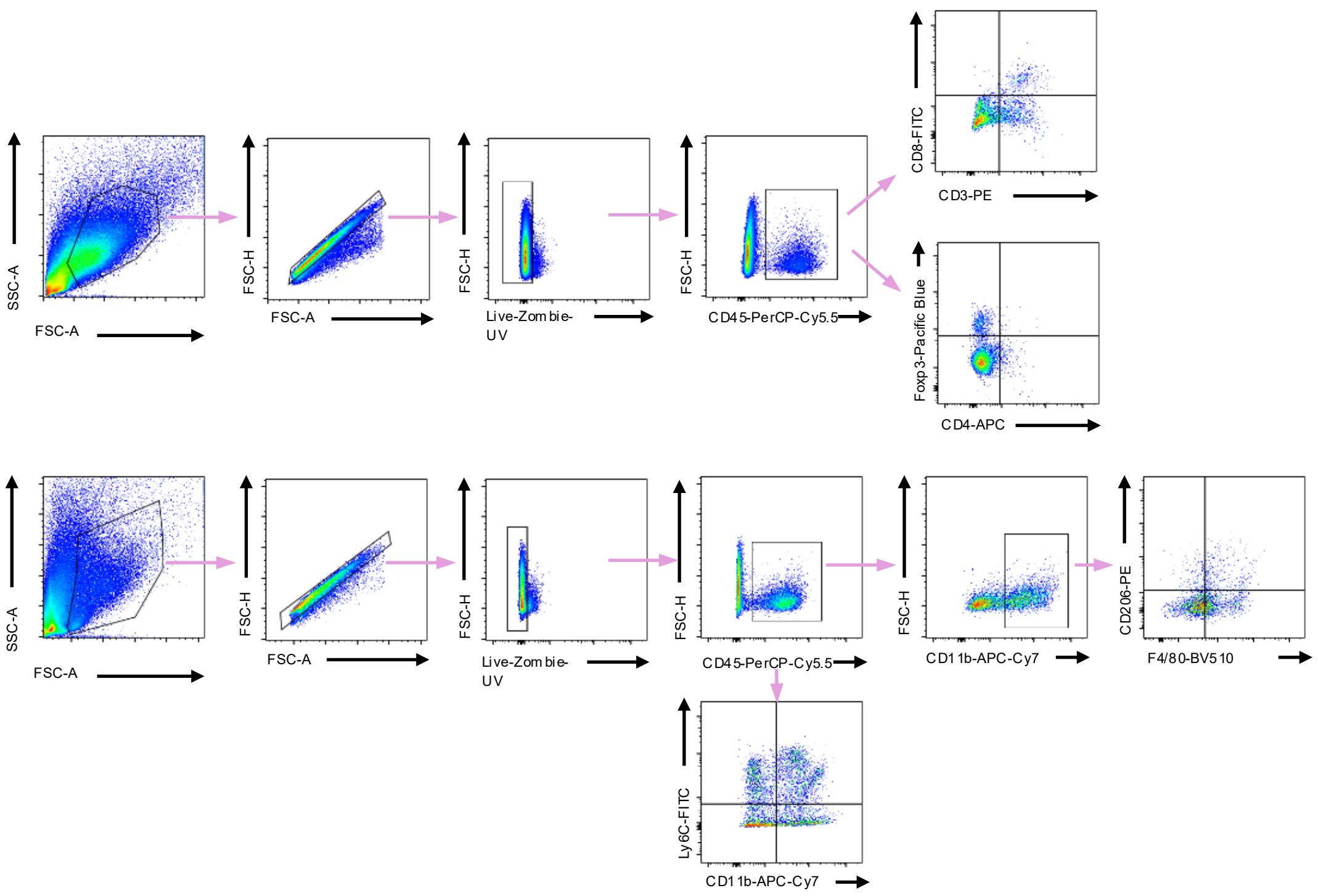

Suppl. Fig. S2. Flow cytometry gating strategy
