# Supplementary material for: Apoptotic cell overload drives multinucleated giant cell formation after neoadjuvant chemotherapy in colorectal cancer: molecular mechanisms and tumor-suppressive effects
Source: Cell Death Dis. 2026 Oct 5;17(1):907. doi: 10.1038/s41419-026-09320-w (PMC13694713; doi:10.1038/s41419-026-09320-w)

Fig1G

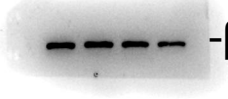

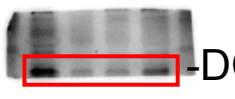

Fig4A

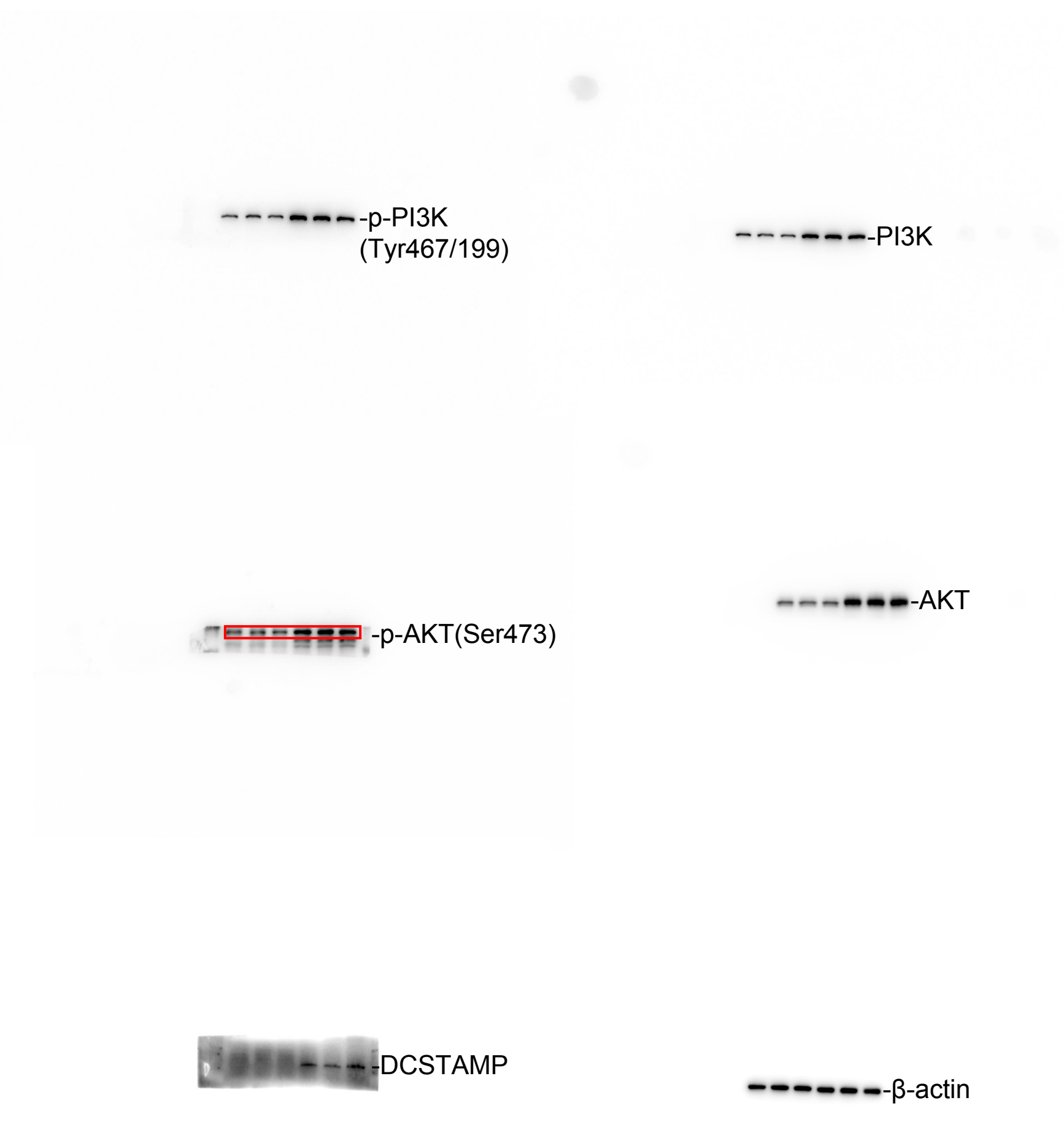

Fig4B

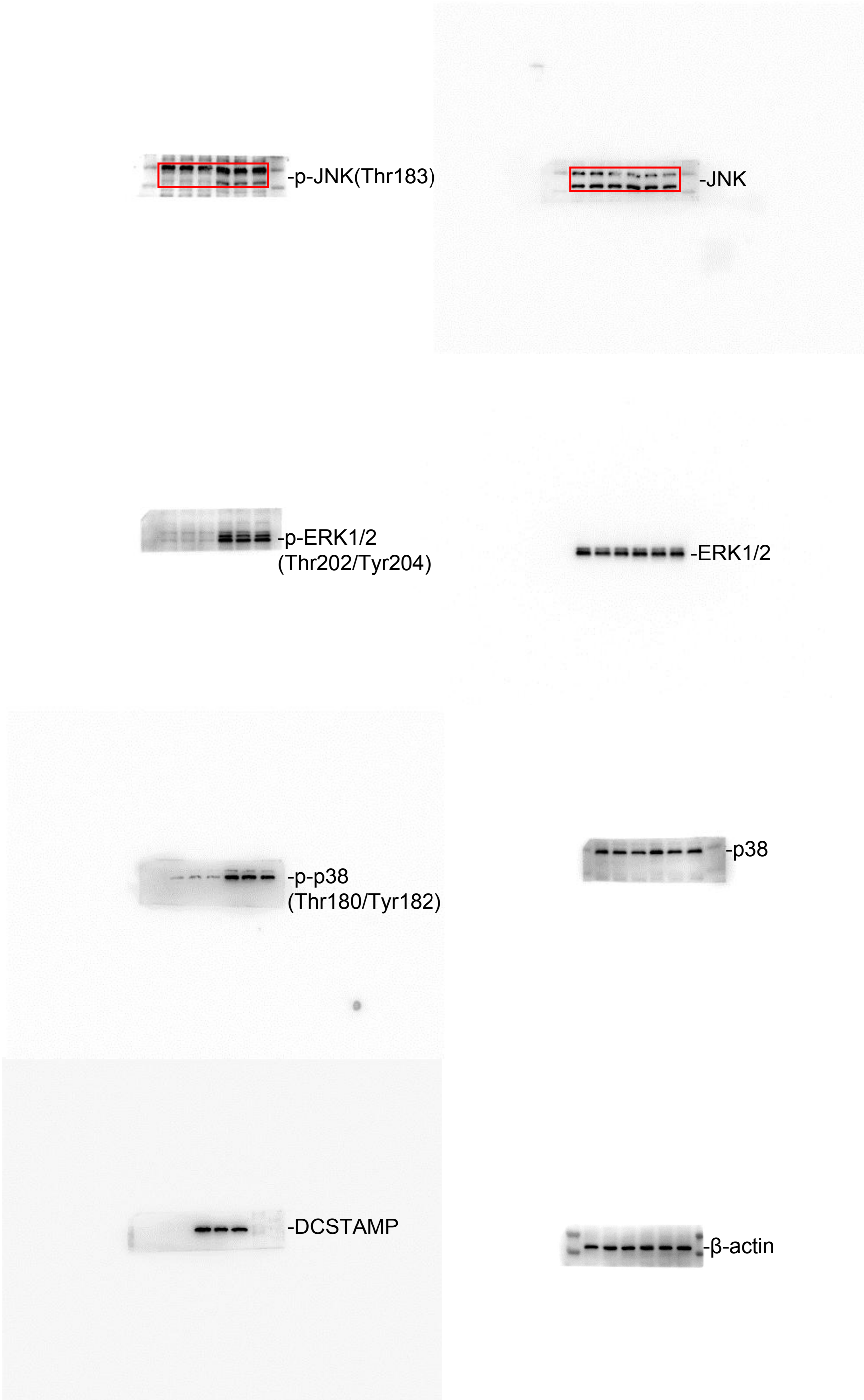
-p-JNK(Thr183)
-JNK
-p-ERK1/2
(Thr202/Tyr204)
-ERK1/2
-p-p38
(Thr180/Tyr182)
-p38
-DCSTAMP
-β-actin

Fig4C

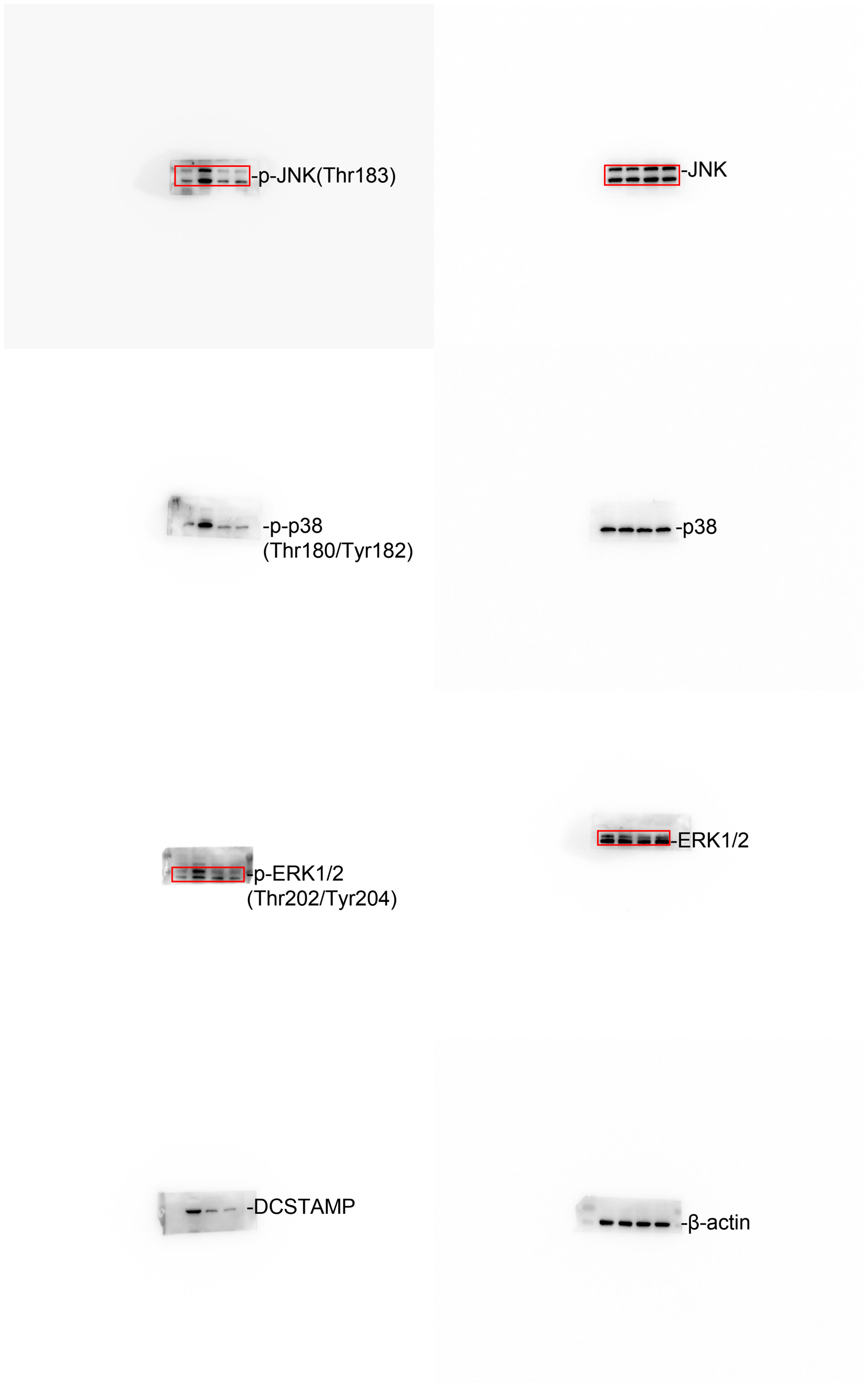
-p-JNK(Thr183)
-JNK
-p-p38
(Thr180/Tyr182)
-p38
-p-ERK1/2
(Thr202/Tyr204)
-ERK1/2
-DCSTAMP
-β-actin

Fig4D

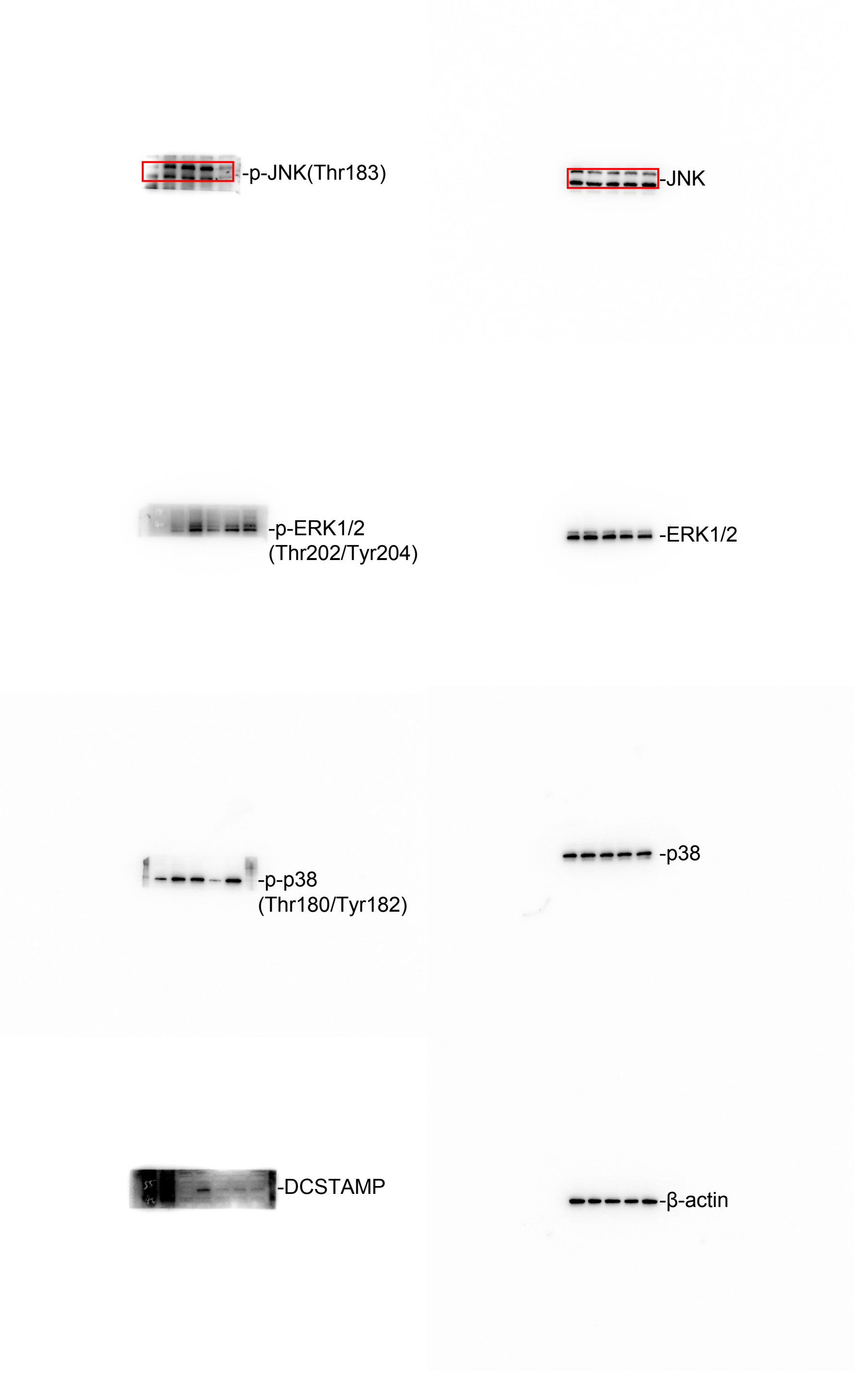
-p-JNK(Thr183)
-JNK
-p-ERK1/2
(Thr202/Tyr204)
-ERK1/2
-p-p38
(Thr180/Tyr182)
-p38
-DCSTAMP
-β-actin

Fig4F

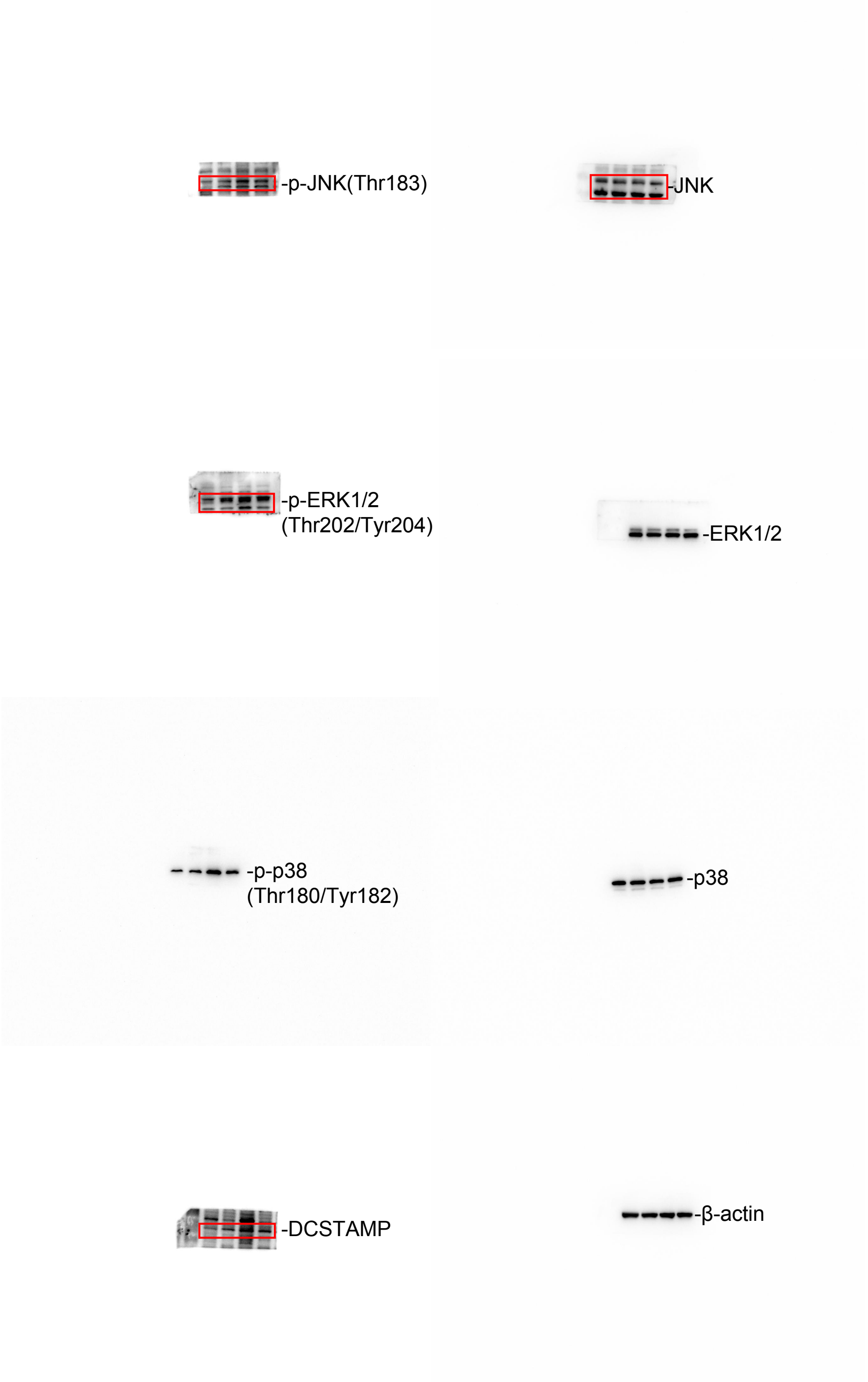
-p-JNK(Thr183)
-JNK
-p-ERK1/2
(Thr202/Tyr204)
-ERK1/2
-p-p38
(Thr180/Tyr182)
-p38
-DCSTAMP
-β-actin

Fig5G

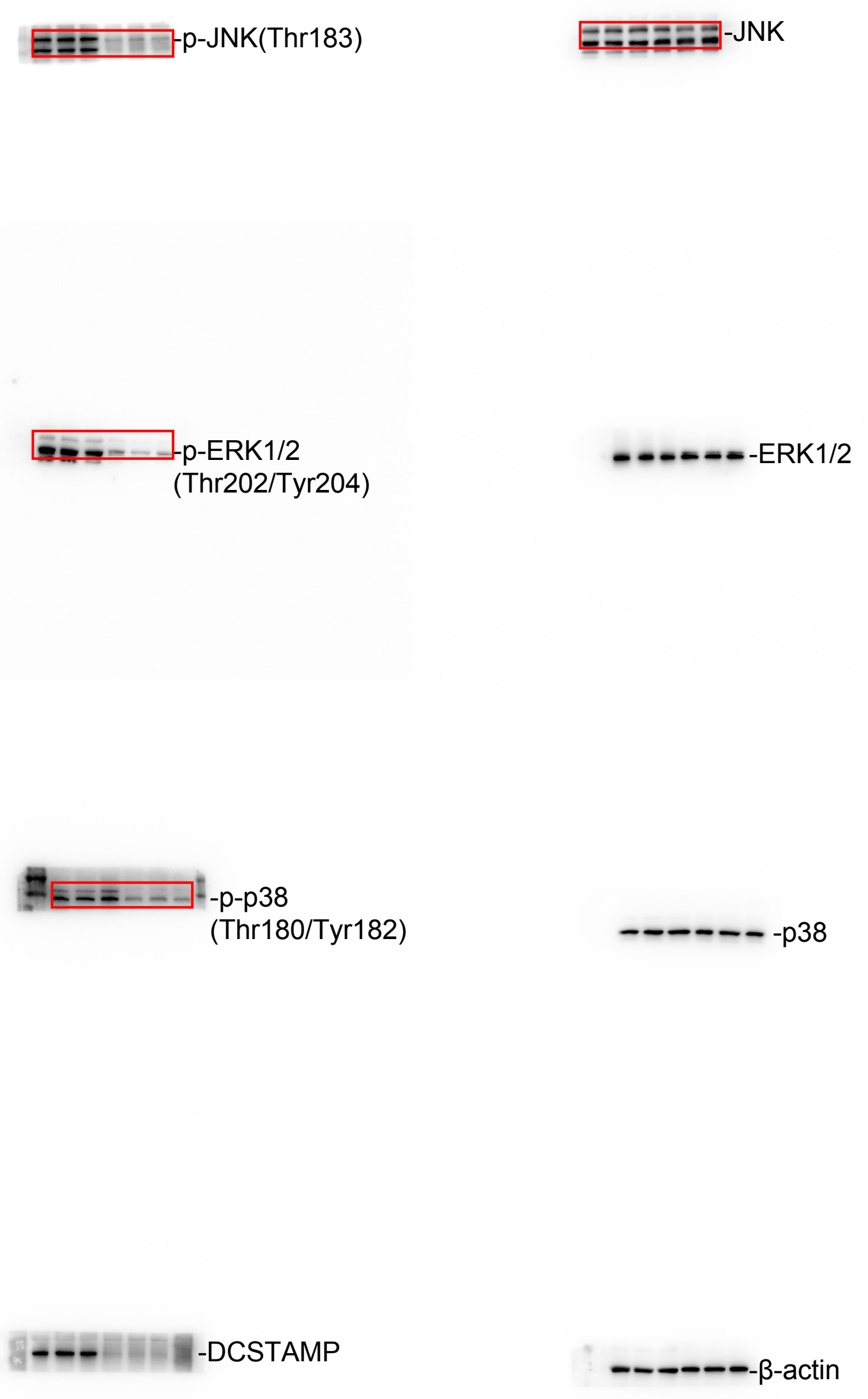
-p-JNK(Thr183)
-JNK
-p-ERK1/2
(Thr202/Tyr204)
-ERK1/2
-p-p38
(Thr180/Tyr182)
-p38
-DCSTAMP
-β-actin

Fig6B

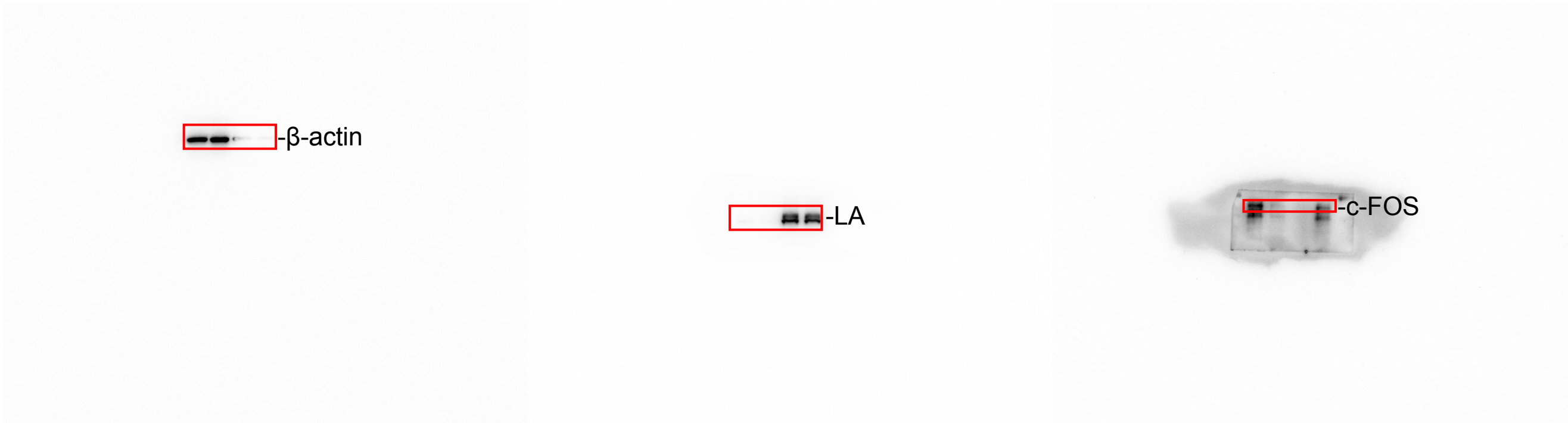

Fig6D

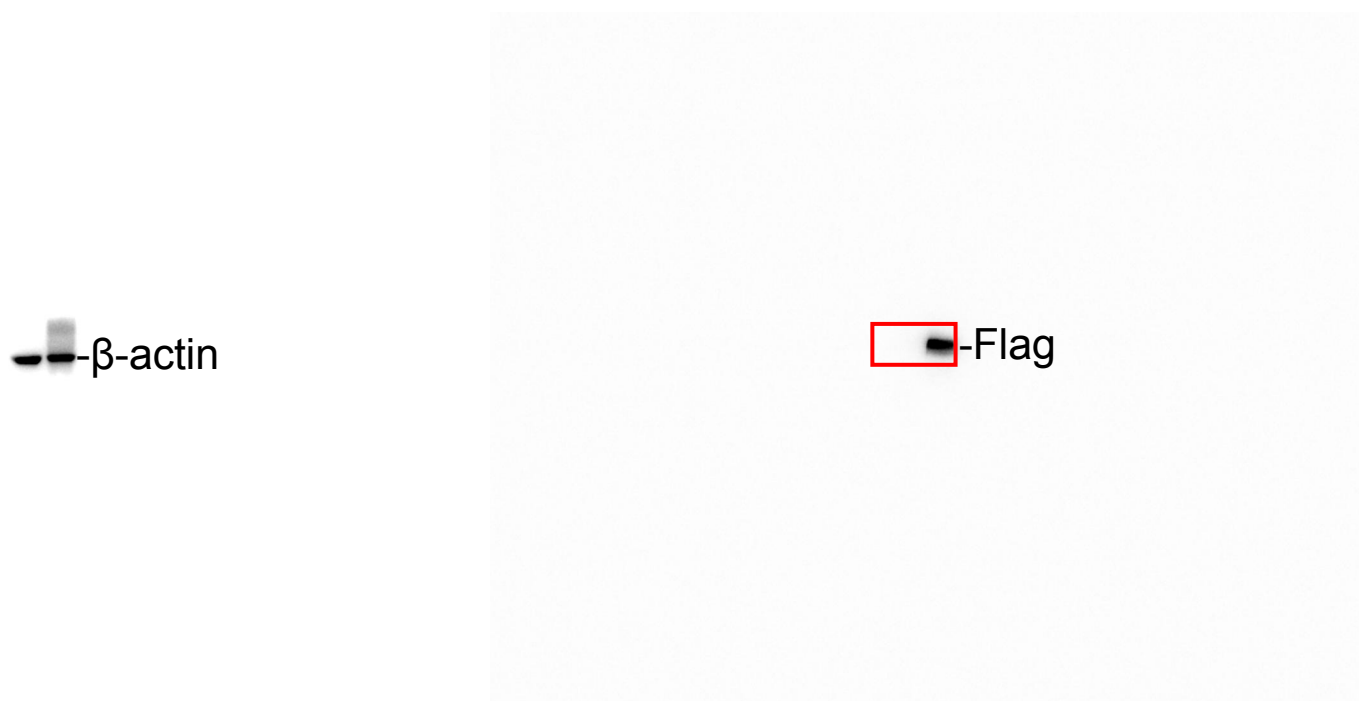

Fig7A

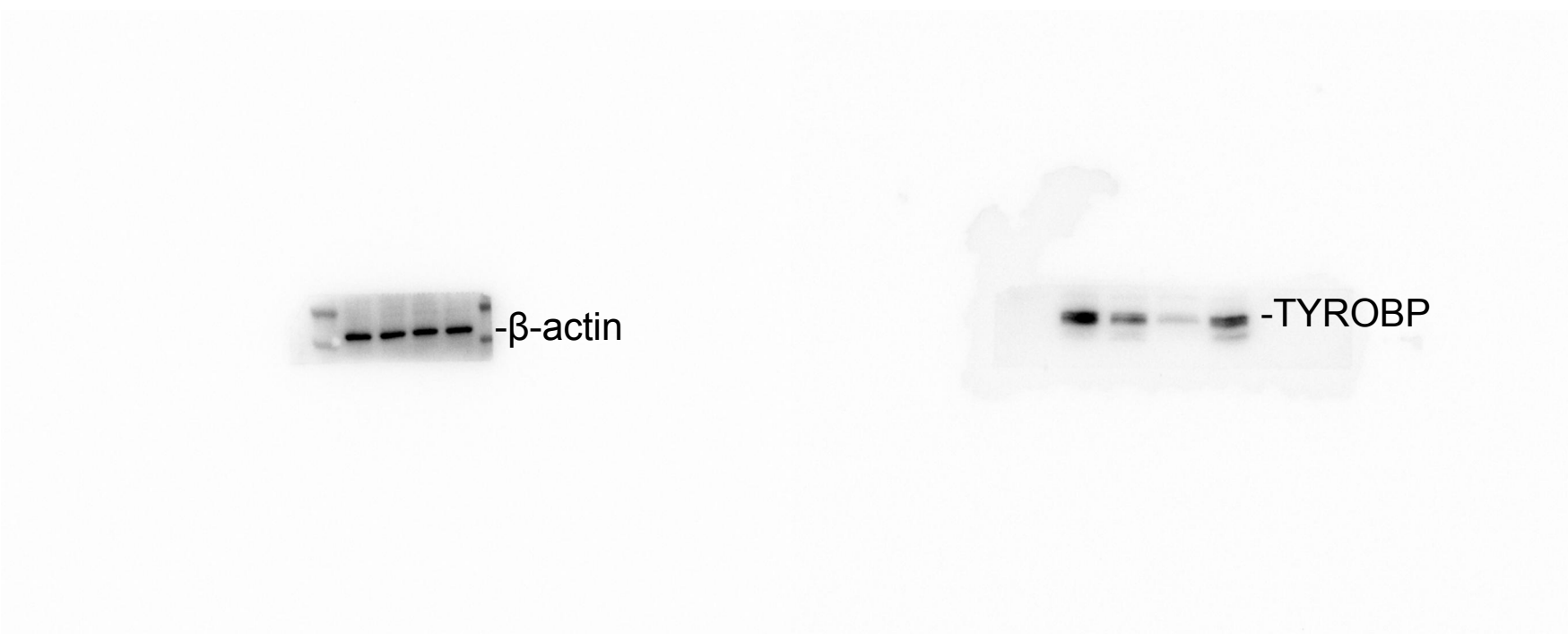

Fig7G

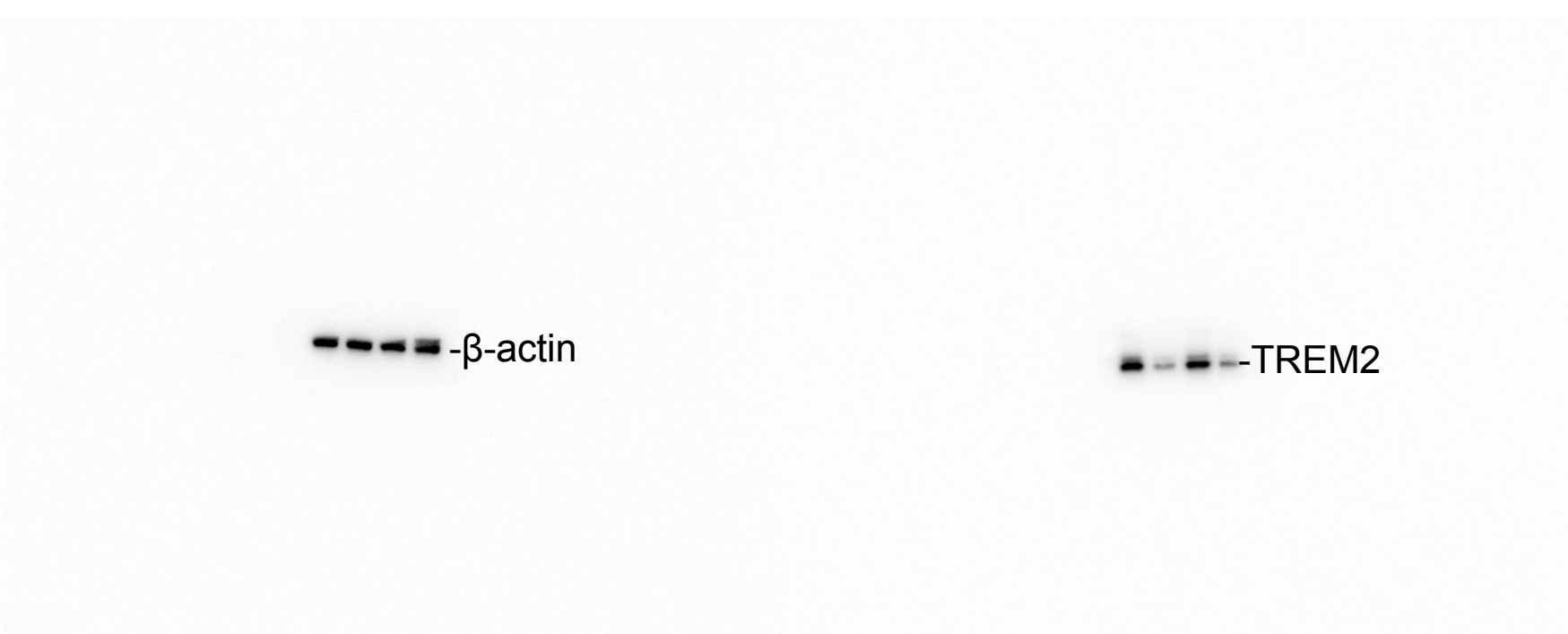

Fig11C

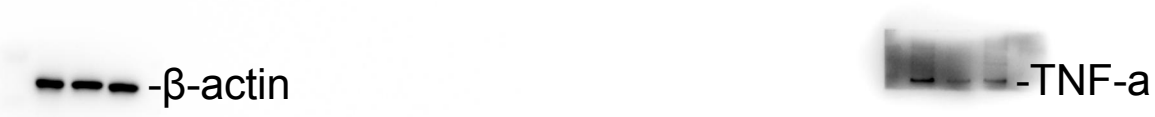

Supplement: Supplementary file 2 [file 41419_2026_9320_MOESM3_ESM.pdf]
